# Brine Enriched with Olive Wastewater Phenols: A Green Strategy to Reduce Nitrites in Cooked Ham

**DOI:** 10.3390/antiox14091124

**Published:** 2025-09-17

**Authors:** Dario Mercatante, Stefania Balzan, Sonia Esposto, Sara Barbieri, Federico Fontana, Luca Fasolato, Vincenzo De Rosa, Maurizio Servili, Agnese Taticchi, Enrico Novelli, Maria Teresa Rodriguez-Estrada

**Affiliations:** 1 Department of Agricultural and Food Sciences, Alma Mater Studiorum—University of Bologna, 40127 Bologna, BO, Italy; dario.mercatante2@unibo.it (D.M.); sara.barbieri@unibo.it (S.B.); vincenzodr91@outlook.it (V.D.R.); maria.rodriguez@unibo.it (M.T.R.-E.); 2 Department of Comparative Biomedicine and Food Science, University of Padova, 35020 Legnaro, PD, Italy; stefania.balzan@unipd.it (S.B.); federico.fontana@unipd.it (F.F.); luca.fasolato@unipd.it (L.F.); enrico.novelli@unipd.it (E.N.); 3 Department of Agricultural, Food and Environmental Sciences, University of Perugia, 06126 Perugia, PG, Italy; sonia.esposto@unipg.it (S.E.); maurizio.servili@unipg.it (M.S.); 4 Interdepartmental Centre for Industrial Agrofood Research, Alma Mater Studiorum—University of Bologna, 47521 Cesena, FC, Italy

**Keywords:** cooked ham, olive oil mill wastewater, biophenols, physico-chemical stability, lipid oxidation, protein oxidation, sensory properties, circular economy

## Abstract

This study aimed to evaluate the effects of brine enriched with an olive vegetation water (OVW) extract on the physico-chemical, oxidative, and sensory characteristics of cooked ham during storage, as a strategy to partially or totally replace nitrites. Four brines formulated with different concentrations of nitrites in combination with 200 mg of OVW extract/kg product were tested; the cooked ham samples were sliced, placed in trays, packed in a protective atmosphere, and monitored for 30 days at 4 °C. The results showed that phenolic compounds derived from OVW effectively reduced lipid and protein oxidation, limiting the formation of secondary oxidation products such as thiobarbituric acid reactive substances, volatile aldehydes, and cholesterol oxides. Sensory analysis confirmed that the extract did not negatively affect the organoleptic properties of the ham, while also helping to preserve color stability. These findings suggest that brine enriched with OVW phenols can be a promising green strategy to reduce nitrites in cooked ham, which also promotes the sustainable valorization of olive oil by-products.

## 1. Introduction

Cooked ham is one of the most popular meat products worldwide. Among extra-European countries, the United States has the highest pork meat consumption per capita, including cooked ham, with a total of 22.58 kg in 2024 [1]. Regarding European countries, cooked ham is the top-selling cured meat product in France, accounting for nearly a quarter of the national consumption of cured meat products [1]. In Italy, cooked ham turns out to be the most appreciated cured meat product, representing about 27.8% of total cured meats [2], of which a large amount (17,743 tons with a value of 148.5 million EUR; [2]) is exported. In particular, there are a wide variety of cooked hams, which are typically categorized based on several attributes like the type of raw material used in the processing, the brine ingredients (e.g., polyphosphates, starches, carrageenan, and soybean proteins), the technological yield (85–110%), and the presentation of the ham (boneless, bone-in, pieces, whole legs, etc.). Italian cooked ham is classified into three distinct quality categories according to Italian law: high-quality cooked ham, select cooked ham, and cooked ham [3].

The production process of cooked ham involves several stages, including trimming and cutting pig muscles into pieces, injecting brine into the pieces, tumbling the brine-injected pieces, filling the tumbled pieces into molds or casings, cooking, and cooling [4]. Besides containing water and salt, the brine may also include sugars, maltodextrins, proteins of different origins, starch, spices, flavorings, and the additives allowed by Commission Regulation (EU) 2023/2108; the latter lays down the specifications for nitrite salts, whose employment in cooked ham is currently highly recommended in Italy. The preservation of the quality and safety of cooked ham during storage are based on a complex interplay of microbiological, biochemical, and chemical processes. Notably, lipid, protein, and pigment oxidation stand out as key events that lead to quality defects; therefore, the incorporation of nitrites in the brine formulations is a key factor to mitigate such risks. At the same time, nitrites exert relevant antimicrobial action against *C. botulinum*. The presence of these preservative agents, however, is the subject of heated debates, both within the scientific community and among consumers. This concern was raised following the 2015 classification by the International Agency for Research on Cancer (IARC), which categorized processed meats containing added nitrate/nitrite salts as carcinogenic to humans (Group 1). These additives can lead to the formation of N-nitroso compounds (NOCs) and nitrosamines, which may be generated during processing, cooking, and storage. Their accumulation and subsequent ingestion have been associated with an increased risk of colorectal cancer [5]. Therefore, regardless of the technological advantages, a reduction in nitrites in cooked meat products has become a priority matter for both industries and consumers. To this purpose, the recent Commission Regulation (EU) 2023/2108 has decreased the maximum allowed levels of nitrites in cooked meat products from 150 (on salt basis) to 80 mg/kg (on NO_2_^−^ ion basis), which will come into force on 9 October 2025. However, their partial or complete replacement with a single compound is a great challenge due to the multifunctional characteristics of nitrites. One possible alternative to the use of this chemical additive is the addition of natural vegetable extracts that contain several bioactive compounds, which could help stabilizing the cooked meat products [6,7]. Phenolic compounds, abundant in plant extracts and widely present in plant-based foods, are well known for their antioxidant and antimicrobial properties [8]. In addition to these effects, they inhibit nitrosation reactions by scavenging reactive nitrogen species and chelating transition metals that catalyze nitrosation pathways [9]. This dual functionality, counteracting N-nitroso compound formation while providing health-promoting effects such as anti-inflammatory, antimicrobial, and cardioprotective activities, makes polyphenols particularly attractive for food applications [10,11]. In meat systems, polyphenols can directly interfere with nitrite chemistry through multiple mechanisms. They may reduce nitrite to nitric oxide, thereby decreasing residual nitrite available for nitrosation reactions, scavenge reactive nitrosating species (such as N_2_O_3_), and inhibit the conversion of secondary amines into carcinogenic N-nitrosamines [12,13]. Certain phenolic structures can also form stable complexes with amines, preventing their interaction with nitrosating agents. These molecular interactions complement their role in limiting lipid and protein oxidation, reducing volatile aldehyde formation, and preserving sensory and technological quality in meat products [14].

One interesting source of hydrophilic phenols is the olive vegetation water (OVW), which is a by-product generated during the mechanical extraction of virgin olive oil [15]. The valorization of OVW represents a multifaceted opportunity to address environmental challenges while implementing the economic potential of this by-product. In fact, OVW has a high organic load and acidity, so its disposal poses significant environmental concerns [16]. However, through innovative technologies and sustainable practices like selective membrane filtration [17], phenolic compounds can be recovered from OVW, thus obtaining high-value extracts that can be used in different industrial sectors and contributing to the circular economy. Hydrophilic phenols from OVW (primarily secoiridoids and their derivatives [18]) possess notable antioxidant, antimicrobial, and anti-inflammatory properties [10,11,19]. Owing to these bioactivities, they have been proposed as natural alternatives for the formulation of cooked meat products to reduce or replace added nitrite salts [20]. Therefore, the aim of this study was to evaluate the effect of brine enriched with an OVW extract on the physico-chemical and sensory attributes of cooked ham during storage, as a strategy to replace (partially or totally) nitrites in this meat product.

## 2. Materials and Methods

### 2.1. OVW Phenol Extract

A crude extract rich in phenols (PE) was obtained from fresh OVW of olives harvested in Umbria (Central Italy) from trees of the Moraiolo cultivar by a 3-step membrane filtration of fresh OVW, as previously reported by Servili et al. [17]. To obtain a more stable and easier-to-use powder formulation, PE was added with maltodextrin (Glucidex 19, Lestrem, France) (1:1, d.w.), as a support, and then spray-dried [15].

### 2.2. Preparation of Phenol-Enriched Cooked Ham

Cooked ham was prepared using boneless and skinless fresh pork thighs of Italian heavy pig (Brunello Domenico s.r.l., Bassano del Grappa, Italy). The basal brine was prepared as follows: sodium chloride (1.5%), glucose (0.2%), ascorbic acid (0.02%), flavors (0.15%) (Metroz Essences S.p.a., Cologno M.se, MI, Italy). Diverse levels of sodium nitrite (Morgan, Tavarnelle Val di Pesa, FI, Italy) and PE were added to the brine to achieve four product formulations: (1) control (0.39% maltodextrin (Glucidex 19, Roquette Freres, Lestreme, France) + 150 mg of NO_2_/kg); (2) S1 (200 mg of PE/kg + 150 mg of NO_2_/kg); (3) S2 (200 mg of PE/kg + 35 mg of NO_2_/kg); (4) S3 (only 200 mg of PE/kg). Three independent replicates were prepared for each formulation.

The meat was injected with the brine using a manual injector, Günther Maschinenbau PP3 (Dieburg, Germany). Each thigh was then placed inside a plastic bag (45 × 70 cm and 140 μm thickness, Morgan, Tavernelle Val di Pesa, Italy) and vacuum-packed to prevent losses and contamination during churning. This phase lasted a total of 70 min, alternating between 5 min of motion and 5 min of rest. Once churning was completed, the packaged thighs were allowed to rest in the cold room overnight. They were then removed from the bags, placed inside aluminum pressure molds, and weighed before being cooked. The thighs were then cooked in a steam oven (ChefTopTM Unox, Cadoneghe, Italy) with 100% humidity and gradually increasing temperature. The cooking time varied depending on when the core temperature of the product was reached according to the cooking program shown in Table 1. Once cooking was finished, the hams were cooled in a blast chiller (Attila GN I/I Tecnodom, Vigodarzere, Italy) until the core temperature reached 4 °C. Hams were then refrigerated for one day at 4 °C, and the post-cooking weight was determined. Subsequently, the cooked thighs were sliced, placed in trays (70 g), and packed in a protective atmosphere (Alipak 120–80% N_2_ and 20% CO_2_-ORVED VGP packaging machine, Musile di Piave, Italy). The trays were then stored in a refrigerated display counter set at 4 °C (Carel Industries Spa, Brugine, Italy) with lighting from 9 am to 8 pm, followed by 13 h of darkness. The trays were sampled at time 0 (production date) and after 15 and 30 days.

### 2.3. pH, Activity Water, Proximate Composition, and Microbial Target of Cooked Ham

The pH was measured after sample homogenization in distilled water (1:10, *w*/*v*) using a Portamess pH-meter (Knick 910, Berlin, Germany) equipped with an INLAB 427 electrode (Mettler Toledo, Urdof, Switzerland). Water activity was determined using a hygrometer AquaLab 4 TEV (Decagon Devices, Pullman, WA, USA), according to Boschetti et al. [21]. Moisture, crude protein, and salt (Volhard method) were determined according to AOAC [22], while the fat percentage was determined according to Folch et al. [23]. Microbial analyses were performed according to Fasolato et al. [24] (see supplementary materials). Measurements were performed in triplicate.

### 2.4. Phenols Analysis

Five grams of cooked ham were mixed with 50 mL of methanol–water (80:20, *v*/*v*) containing 20 mg/mL of butylated hydroxytoluene (BHT) and 0.2% trichloroacetic acid (TCA) 2 M. The solution was then homogenized for 1 min, and then centrifuged at 9000 rpm for 10 min. After collecting the supernatant, the procedure was repeated on the pellet. The supernatants were combined and concentrated to a volume of 20 mL. Ten mL of this extract were purified by C18 solid phase extraction (SPE) [25]. The purified extract was analyzed using high-performance liquid chromatography coupled with a diode array detector (HPLC-DAD, Agilent Technologies, mod. 1100 Series, Santa Clara, CA, USA), according to the analytical protocol described by Selvaggini et al. [26]. For the PE analysis, 50 mg of the extract were dissolved in 10 mL of a methanol–water solution (80:20, *v*/*v*), filtered through a 0.2 μm polyvinylidene fluoride (PVDF) syringe filter (Agilent Captiva, Agilent Technologies), and subsequently injected into the HPLC-DAD system. Measurements were performed in triplicate.

### 2.5. Protein Oxidation

#### 2.5.1. Carbonyl Compounds

Carbonyl content and protein concentration were measured according to Zakrys et al. [27]. Four aliquots of 2 g of each sample were homogenized in 20 mL of 0.15 M KCl buffer at 13,500 rpm for 15 s (Ultra Turrax T25, Staufen, Germany); three aliquots were used for the carbonyl measurement, while the fourth was employed for the estimation of protein concentration. The homogenate (45 μL) was mixed with 1 mL of 10% TCA in a 2 mL vial and centrifuged at 5000× *g* for 5 min (Centrifuge 5425, Eppendorf, Hamburg, Germany); the supernatant was then removed. A protein pellet was added with 1 mL of 0.2% 2,4-dinitrophenyl hydrazine (DNPH) in 2 M HCl for carbonyl measurement, whereas 1 mL of 2 M HCl was added for the evaluation of protein content. After 1 h incubation in the dark at room temperature, 1 mL of 10% TCA was added; the samples were then vortexed and centrifuged at 5000× *g* for 5 min, and the supernatant was cautiously discharged. The pellet was washed three times with 1 mL of ethanol–ethyl acetate (1:1, *v*/*v*), shaken, and centrifuged at 5000× *g* for 5 min, after which the pellet was dried under nitrogen flow. The pellet was finally solubilized in 6 M guanidine hydrochloride dissolved in 0.02 M sodium phosphate buffer, pH 6.5, shaken, and centrifuged at 5000× *g* for 2 min. Carbonyls were measured at 370 nm (Jasco 7800 UV/VIS spectrophotometer, Cremella, Italy) on the DNPH-treated samples, and their concentration was calculated using the molar extinction coefficient of 21 mM^−1^ cm^−1^. Protein concentration was calculated using a standard curve of bovine serum albumin solubilized in 6 M guanidine hydrochloride (concentration range: 0.75–6 μM) and measured at 280 nm. A blank was prepared, replacing the homogenate with 1 mL of 10% TCA. The carbonyl content was expressed as nmol DNPH/mg protein.

#### 2.5.2. Sulfhydryl Groups

Total sulfhydryl (SH) content was determined according to Eymard et al. [28], with slight modifications. Four aliquots of 2 g of each sample were homogenized with 20 mL of 0.05 M sodium phosphate buffer, pH 7.2, at 13,500 rpm for 15 s, using an UltraTurrax T25 (Staufen, Germany). Again, three of them were used for the SH measurement, whereas the fourth one was utilized for the quantification of the protein concentration using the Lowry protein assay (Total protein kit, Sigma-Aldrich, Milano, Italy). The homogenate (200 μL) was transferred to a 2 mL vial, and 1800 μL of phosphate buffer 0.05 M, pH 7.2, that contained 0.6 M NaCl, 6 mM EDTA, and 8 M urea were added. After centrifugation (5 min at 5000× *g*, Centrifuge 5425, Hamburg, Germany), 26 μL of 0.01 M 5,5′-dithio-bis-(2-nitrobenzoic acid) in 0.05 M sodium acetate were added to 1.95 mL of supernatant and incubated in a water bath (M428-BD, MPM Instruments s.r.l., Bernareggio, Italy) at 40 °C for 20 min. A blank was prepared, replacing the homogenate with 200 μL of 0.05 M phosphate buffer, pH 7.2. The absorbance was measured at 412 nm. SH concentration was expressed in nmol SH/mg protein, which was calculated using a molar extinction coefficient of 14,100 M^−1^ cm^−1^ [29].

### 2.6. Lipid Extraction

Fat extraction was performed on 10 g of cooked ham, added with 1 mg of 5α-cholestane (internal standard for quantification of the main lipid classes) (Sigma Chemical, St. Louis, MO, USA), as reported by Boselli et al. [30]. After the extraction, the fat content was gravimetrically determined. Three independent extractions were performed for each sample.

### 2.7. Determination of Main Lipid Classes

The quali-quantitative profile of the main lipid classes (including free fatty acids (FFA), monoacylglycerols (MAG), free sterols (STE), diacylglycerols (DAG), esterified sterols (E-STE), and triacylglycerols (TAG)) was determined by gas chromatography-flame ionization detection (GC-FID; GC-2010 Plus, Shimadzu, Kyoto, Japan), according to the methods described by Luise et al. [31]. A 20 mg aliquot of the lipid extract was dissolved in 1 mL of *n*-hexane and analyzed by GC-FID. The quantification of the lipid classes was performed using the internal standard method; response factors were previously determined using commercial standards for each lipid class. Results were expressed as g/100 g of total lipids. All analyses were performed in triplicate.

### 2.8. Determination of Total FA

After a previous methylation and transmethylation, the composition of total fatty acids was determined on 20 mg of lipid extract by GC-FID (GC8000 Fisons Instruments, Milan, Italy) [32]. FAME quantification was performed using tridecanoic acid methyl ester (0.6364 mg) as an internal standard and expressed as g/100 g of FAME. All analyses were performed in triplicate.

### 2.9. Determination of Thiobarbituric Acid Reactive Substances (TBARs)

Secondary lipid oxidation was evaluated using TBARs [33] as an indicator. Two g of each cooked ham sample were utilized for this spectrophotometric analysis (spectrophotometer model V-550, Jasco, Tokyo, Japan) with absorbance readings taken at 530 nm. Quantification of TBARs was achieved through a calibration curve based on 1,1,3,3-tetramethoxypropane standards (concentration range: 0.045–0.113 μg/mL; equation: y = 0.0077x + 0.0072, r^2^ = 0.9998), and the results were reported as mg MDA/kg meat. All analyses were performed in triplicate.

### 2.10. Determination of Cholesterol and Cholesterol Oxidation Products (COPs)

For the determination of total cholesterol and cholesterol oxidation products (COPs), betulinol (Sigma Chemical, St. Louis, MO, USA) and 19-hydroxycholesterol (Steraloids, Newport, RI, USA) were added as internal standards, respectively, to 200 mg of lipid extract. Samples were subjected to cold saponification and subsequently purified using aminopropyl solid-phase extraction (SPE) cartridges, as described by [32]. Following silylation, the analytes were injected into a Fast gas chromatograph coupled to a mass spectrometer (Fast GC/MS; GCMS-QP2010 Plus, Shimadzu, Kyoto, Japan), according to the analytical conditions reported by Cardenia et al. [34]. Mass spectra were acquired in full scan mode (total ion current, TIC) and further analyzed using selected ion monitoring (SIM) based on highly abundant characteristic ions. Quantification of total cholesterol, individual COPs, and total COPs was performed using compound-specific calibration curves, with results expressed as mg/kg of meat. All analyses were performed in triplicate. The cholesterol oxidation rate (%OR) was also calculated according to the method described by Cardenia et al. [32].

### 2.11. Volatile Organic Compounds (VOCs) Analysis

Headspace VOCs analysis was performed using solid phase microextraction–gas chromatography/mass spectrometry (SPME-GC/MS, GC 7890B/MSD 5977B XTR Agilent Technologies, Santa Clara, CA, USA). In a 20 mL vial, 50 μL of internal standard (4-methyl-2-pentanol, 3 mg/kg) and 2 mL of a saturated aqueous NaCl solution were added to 1 g of homogenized ham. The vial was subsequently tightly sealed with a PTFE (polytetrafluoroethylene) septum and placed in the autosampler. VOCs analysis was performed using the same equipment, analytical conditions, and methodology as described by Dottori et al. [35]. VOCs identification was carried out by comparing the obtained mass spectra and retention times with those of pure analytical standards and with the NIST 2014 library spectra. The quantification of VOCs was calculated by comparing each ion peak area with the internal standard ion peak area (4-methyl-2-pentanol), as reported by Xiao et al. [36], and expressed as µg of VOC/kg of freshly weighted (f.w.) ham.

### 2.12. Physical Analysis

#### 2.12.1. Instrumental Color Measurement

Color changes (CIE L*a*b* system) during storage were checked by a visible spectrophotometer (CM-508d, Minolta Camera Co., Osaka, Japan) with D65 as light source and 10° observed angle (CIE Publication N. 15.2., 1986). Delta E designated the amount of difference in L*, a*, and b* color space and was calculated using the color values measured on day 0 and the readings on days 15 and 30 of shelf life as follows: ΔE = √[(Lx − L0)^2^ + (ax − a0)^2^ + (bx − b0)^2^)] where x indicated the day of shelf life [37].

#### 2.12.2. Image Analysis

The visual analyzer VA400 IRIS (Alpha MOS, Toulouse, France) was applied for visual assessment (color) and to track color changes in cooked ham samples over time, as described by Barbieri et al. [38]. This imaging system was equipped with a high-resolution CCD (charge-coupled device) camera (resolution 2592×1944p), combined with Alphasoft software (version 14.0) for system monitoring, data acquisition, and multivariate statistics processing. Each measurement (picture) was performed in triplicate.

### 2.13. Sensory Analysis

All the panelists who participated in this study were part of a panel previously trained on the product under examination (as part of the Italian program FARB—Financing of Alma Mater Studiorum, University of Bologna for basic research; Line of Action 2—Project Meating “Sensory and fast instrumental analyses of meat and meat products: An integrated approach for quality control and communication” [38]). They were aware and informed about procedures and risks. Specifically, the panelists were informed that the project aims were to promote the valorization of by-products of the mechanical extraction process of olive oil as sources of high-value molecules and that they would taste new foods with higher functionality, improved by introducing a phenolic extract obtained from OVWs as a preservative agent for shelf-life extension. All the panelists gave their informed consent for inclusion before they participated in the study.

#### 2.13.1. Descriptive Analysis

The sensory profile of all samples at the 3 different storage times was evaluated by a panel of nine fully trained judges of both genders, aged between 20 and 65 years. For the sensory characterization, a conventional profiling method was applied [39]. The sensory attributes, their definition, the profile sheet, the sample preparation, and the test conditions were the same as described by Barbieri et al. [38] with some adaptations. In fact, considering that this study aimed to discriminate between different formulations (sample C and treated samples) and to evaluate changes during storage, the presence of anomalies (olfactory, gustatory, and visual) was also evaluated. Moreover, among appearance descriptors, only pink intensity was taken into consideration. Results were expressed as the mean of three replicates.

#### 2.13.2. Discriminant Test

The triangle test was applied to identify sensory differences between the control sample (C) and the treated samples just produced (T0) and after different storage times (i.e., 15 (T1) and 30 (T2) days). The test was conducted on three different days involving 44 (first day), 40 (second day), and 29 untrained judges, aged between 20 and 65 years, and according to the procedures described by ISO 4120:2007 [40] and the conditions applied by Barbieri et al. [41]. In each session, 6 triads of samples were evaluated.

### 2.14. Statistical Analysis

Chemical and sensory data were processed using XLSTAT 7.5.2 software (Addinsoft, France). The chemical data represent the mean values obtained from independent replicates of each analytical determination. Initially, the normal distribution of the data was assessed (*p* < 0.05) using the Shapiro–Wilk method. Subsequently, the chemical data were subjected to a two-way analysis of variance (ANOVA), with formulation (Form), storage time (St), and their interaction (Form*St) considered as factors. Tukey’s honest significance test was applied at a 95% confidence level (*p* < 0.05) to distinguish means of statistically different parameters. Furthermore, a principal component analysis (PCA) with Varimax rotation was conducted.

## 3. Results and Discussion

### 3.1. Proximate Composition, pH, a_w_, and Microbial Analyses

Formulation and storage did not affect (*p* > 0.05) the proximate composition (Table 2); data displayed the normal variability of the anatomical cut. The moisture content was slightly lower than that of commercially Italian high-quality cooked hams (>67%) [42]. This could be attributed to the absence of ingredients that bind water and limit cooking losses, as well as to the use of MAP packaging that prevented evaporation losses during storage. Cooking weight losses and technical yields further confirmed this trend, as they were higher and lower, respectively, compared to those of commercial products (Table S1). As reported in Table 2, the lipid content of cooked ham ranged from 6.80% to 13.99% and was significantly affected only by formulation. These findings agree with those of Lucarini et al. [43], who reported comparable lipid levels in cooked ham. The substantial variability observed among the data may be attributed to differences in the anatomical and physiological characteristics of the pork thighs used, which inherently vary in fat deposition due to animal-specific factors. A_w_ and pH values (Table 2) were in line with the products marketed in Italy [44], which are known to be insufficient to guarantee the products’ shelf-life [45]. To verify that the addition of OVW phenols would not cause adverse effects on the residual microbial population, especially in the batches without or with reduced nitrite, a few general microbial markers were monitored during cooked ham storage. There was a trend in the reduction in the microbial load over time, without significant differences between control and experimental batches (Table S2).

### 3.2. Evolution of Phenolic Compounds

The PE used in the preparation of the cooked ham contained a total phenolic content of 25.7 mg/g of dried products. The phenolic profile was dominated by 3,4-DHPEA-EDA (oleacein), which accounted for 61.5% of the total, followed by 3,4-DHPEA (hydroxytyrosol, 20.6%), verbascoside (13.2%), and *p*-HPEA (tyrosol, 4.7%). As shown in Table 3, at time 0, in samples S1–S3 (all with 200 mg/kg of phenols), the ham manufacturing processes resulted in a loss of phenolic compounds, with a total phenol reduction varying between 25% and 30% of the initial amount introduced.

The greatest losses were observed in samples S2 and S1, whereas sample S3 exhibited the lowest reduction. 3,4-DHPEA-EDA was the most affected compound during manufacturing, with losses reaching up to 82% in sample S2. In contrast, the concentration of 3,4-DHPEA increased in all PE-enriched samples after the manufacturing process, despite an initial spiked amount of 41.2 mg/kg. However, a portion of the added phenols was also lost during storage. Specifically, total phenol content in S1 samples decreased by 32% and 52% after 15 and 30 days, respectively, while more pronounced reductions were observed in S2 (47% and 66%) and S3 (28% and 58%) samples (Table 3). Among the individual compounds, 3,4-DHPEA-EDA exhibited the greatest instability, becoming already undetectable in all PE-enriched samples after 15 days of storage. As observed and explained in other experiments (e.g., in a fermented functional milk [46] and in fresh pork sausages [47], 3,4-DHPEA-EDA underwent hydrolysis during ham manufacturing and cooking, releasing free 3,4-DHPEA. As described by Obied et al. [48], the degradation of this oleuropein derivative involves not only enzymatic and non-enzymatic oxidation, but also the hydrolytic cleavage of the bond linking the phenolic moiety to decarboxymethyl elenolic acid. The evolution of phenolic compounds in S1, S2, and S3 cooked ham supports the hypothesis that their presence originates from the hydrolytic degradation of oleuropein derivatives occurring during processing and storage [49].

The oxidative degradation of these two phenols (3,4-DHPEA-EDA and 3,4-DHPEA) has been identified as the predominant driver of their decline [48,50]. The temporal evolution of 3,4-DHPEA appears to be dictated by two concurrent processes: the release of free 3,4-DHPEA following the hydrolysis of 3,4-DHPEA-EDA, and its subsequent depletion via oxidative pathways. During the initial storage period of samples S1, S2, and S3, the involvement of 3,4-DHPEA in oxidative reactions was minimal. Consequently, the dynamic interplay between its release and degradation resulted in a net accumulation of 3,4-DHPEA with respect to the initially added amount. In the later stages of storage, oxidative degradation became the dominant process in PE-enriched cooked ham samples, likely due to the depletion of more reactive phenolic compounds such as 3,4-DHPEA-EDA. In contrast, the concentrations of *p*-HPEA and verbascoside remained relatively stable throughout the storage period, in agreement with previous shelf-life studies on cooked meat products [47]. Finally, it is important to emphasize how the chemical hydrolysis of phenolic compounds in cooked ham plays a significant role in determining the product’s quality and health benefits [51]. While cooking reduces the initial levels of these compounds, some are retained during storage, as shown in Table 3, contributing to antioxidant activity and improved oxidative stability.

### 3.3. Main Lipid Classes

Regarding the main lipid classes (Table 4), TAG were the most abundant, followed by DAG, FFA, and STE. The content of all lipid classes was significantly influenced by formulation, storage time, and their interaction; however, no consistent trend was observed across the samples. These differences may be partially attributed to the formulation of the cooked hams, as they were prepared using pork thighs from different animals. Additionally, variations in leg trimming, performed manually prior to ingredient addition and cooking, could also influence these parameters depending on the extent of cover fat removal. Moreover, lipid hydrolysis can significantly impact the nutritional quality and sensory characteristics of cooked ham. TAG are broken into FFA, partial glycerides, and glycerol; this can enhance the flavor profile of cooked ham, as FFA are precursors to various volatile compounds that contribute to aroma and taste. The breakdown of lipids can also influence the nutritional profile of cooked ham. The release of FFA can improve the bioavailability of certain nutrients and contribute to the overall health benefits associated with lean meat consumption [52].

### 3.4. Total Fatty Acid Profile

Concerning total FA composition (Table 5), monounsaturated fatty acids (MUFA) were the predominant class (70–75%), followed by saturated fatty acids (SFA, 13–16%) and polyunsaturated fatty acids (PUFA, 12–16%). This FA profile is consistent with that reported by Garbowska et al. [53] for cooked ham. MUFA content was significantly affected by formulation, storage, and their interaction. Specifically, MUFA increased in the C sample over the storage period, whereas a decrease was observed in the PE-enriched samples. In contrast, only the storage time significantly influenced SFA content. PUFA levels were affected by both formulation and the interaction between formulation and storage, even though no consistent trend was noted. It can be observed, however, that the PUFA content of C samples tends to decrease along the shelf-life, while it remains stable or tends to increase slightly in samples where PE had been added. For SFA content, a general decline in their content can be observed in all analyzed samples.

Regarding the ratios of FA classes (Table 5), the *n*-6/*n*-3 ratio ranged from 4.55 to 7.52, in line with values reported by Parrini et al. [54] for cooked pork meat products. This ratio is widely recognized as a relevant nutritional index, as lower values are associated with improved health outcomes. According to Simopoulos [55], an *n*-6/*n*-3 ratio below 4 is considered optimal for a healthy human diet. This ratio was significantly affected by formulation, storage, and their interaction. Its variation appears to be largely driven by the reduced levels of *n*-6 PUFAs observed in samples S2 and S3, which were formulated with 35 and 0 mg NO_2_/kg + 200 mg PE/kg, respectively. The PUFA *n*-6 decrease could be partly due to oxidation, which also affected PUFA *n*-3. In addition to this degradation process, these differences could also be ascribable to the different levels of fat deposition in the thighs used for cooked ham formulation, as well as from the manual trimming operations carried out during sample preparation. On the other hand, the higher amount of NO_2_ (150 mg/kg) added to samples C and S1 allowed them to preserve PUFA from oxidation more efficiently than PE.

The PUFA/SFA ratio is another important index for assessing the nutritional quality of food lipids, with nutritional guidelines recommending a value around 0.4 [56]. In the present study, this ratio ranged from 0.70 to 1.31, in agreement with the values reported by Garbowska et al. [53] for cooked ham. The ratio followed the same pattern as the SFA class, being significantly influenced by storage time, even though no significant interaction was observed between the tested factors.

The UFA/SFA ratio, which serves as an indicator of FA oxidative stability, decreases as UFA undergo oxidation. In this study, the UFA/SFA ratio ranged from 5.40 to 7.80, with storage time being the only factor that significantly influenced this ratio, thus leading to an increase in all samples at T30.

Regarding individual FA, oleic acid (C18:1 *n-9*) was the most abundant (67.16–75.58%), followed by stearic acid (C18:0, 5.22–13.91%) and linoleic acid (C18:2 *n-6*, 8.88–13.74%) (Table S3). These values are consistent with those previously reported by Garbowska et al. [53] for cooked ham. No significant differences in the profile of individual FA were observed between the control and PE-enriched samples throughout the shelf-life period. Minor variations could be partially attributed to matrix variability, likely resulting from differences in the fat content of the pork legs used for cooked ham [53].

### 3.5. Protein Oxidation

Table 6 shows the results of the oxidative deterioration of proteins during storage. Direct oxidation of susceptible amino acid side chains is considered the main chemical mechanism for protein carbonylation in meat [57,58,59]. The increase in protein carbonyl content in meat products during storage is a well-known phenomenon [45], but it can also decrease since carbonyls are not terminal compounds but can be transformed into carboxylic acids and Schiff bases [60]. A significant decrease in carbonyl content was detected in samples S1 and S3 after 15 days of storage, evidencing a prevailing carbonyl conversion trend rather than their accumulation. Conversely, at day 15, samples S2 exhibited an unexpected increase in carbonyls. This phenomenon can be plausibly explained by a short propagation phase of oxidation, likely associated with a faster depletion and/or partitioning of polar phenolic compounds from the lipid phase toward the aqueous protein phase [61,62]. In addition, the carry-over of trace pro-oxidant species, such as hydroperoxides or transition metals from processing, along with slight differences in headspace oxygen availability, may also have contributed [61,62]. These factors are known to trigger batch-specific fluctuations in carbonyl levels before a new oxidative steady state is established.

In addition, the carbonyl content was significantly affected by the use of nitrite and PE, limiting its increase due to the antioxidant activity of the added compounds [45].

The loss of sulfhydryl groups during storage (*p* < 0.001) was 8%, 29%, 48%, and 58% for samples S1, S2, S3, and C, respectively. At the end of storage, the use of PE + 150 mg of nitrites/kg (S1) was the most protective combination. The synergic action against protein oxidation was confirmed in this study; in fact, several studies [45,63] have reported the capacity of phenolic compounds to delay protein oxidation, highlighting a different efficacy among the extracts or essential oils tested. This is due to the type of phenols present, the different antioxidant mechanisms, and the natural variability of the composition [63]. Sulfhydryl groups can also act as reducing agents; in fact, they can react with oxidized phenolic compounds or free radicals, thereby regenerating the antioxidant capacity of the phenols [64]. Moreover, phenols can form reversible non-covalent interactions with proteins through hydrophobic, electrostatic, and hydrogen bonding, which contribute to their antioxidant activity [65].

### 3.6. Lipid Oxidation

Lipid oxidation in the cooked ham samples was monitored by measuring TBARs and COPs, both recognized as indicators of secondary lipid oxidation (Table 6). TBARs values ranged from 0.64 to 3.63 mg MDA/kg of meat. Both product formulation and storage time had a significant impact on this oxidative parameter, with their interaction also being statistically significant. Throughout the shelf-life study, sample S1 exhibited the highest oxidative stability, maintaining TBAR values close to 1.0 mg MDA/kg of meat (threshold value commonly associated with the onset of rancidity in cooked pork products [66]), whereas the control sample displayed significantly higher TBAR levels (<3.80 mg MDA/kg of meat). Samples S2 and S3 followed a similar oxidative pattern, with TBAR values remaining below 1.45 mg MDA/kg of meat. The results obtained, therefore, demonstrate how the PE, in synergy with nitrites, can limit oxidation, as confirmed by the study carried out by Balzan et al. [47], in cooked pork-based sausages formulated with a PE deriving from OVW, where TBARs were lower than 1 mg MDA/kg of meat.

Cholesterol is a key component of cell membranes and, like FA, is also prone to oxidation, primarily due to the presence of a double bond in the B ring. The total cholesterol content (Table 6) ranged from 1163.0 to 1872.6 mg/kg of meat, which is consistent with the values reported by Baggio & Bragagnolo [67] for pork-based cooked meat products. Cholesterol levels were significantly influenced by formulation alone. It is important to note that cholesterol content in pork meat can vary considerably depending on the pig breed [68]. Moreover, the substantial variability observed among the data may be attributed to differences in the anatomical and physiological characteristics of the pork thighs used, which inherently vary in fat deposition due to animal-specific factors.

The total COP content in cooked ham samples ranged from 0.88 to 6.27 mg/kg of meat. Similarly to TBARs, their levels were significantly affected by product formulation, storage duration, and the interaction between these two factors. When looking at the trends of individual samples during storage, it can be noted that the amount of COPs in sample C tended to increase after 15 days of storage, but decreased at the end of storage. On the other hand, a sinusoidal trend can be observed in PE-added samples. In particular, total COPs content in sample S1 tended to decrease at T15 (−40% compared to T0) and increase at T30 (+40% compared to T0). In sample S2, on the other hand, a decrease in total COPs was observed at both T15 and T30 (−75% and −50%, respectively), compared to T0. Finally, in sample S3, COP content did not change up to T15, but increased thereafter (+65% compared to T0). This could probably be due to the fact that the extract was not able to fully penetrate inside the cell membrane, where cholesterol is located, thus not allowing it to exert its antioxidant effect towards cholesterol. In fact, cooked ham is a product that is made from whole muscle fractions, so specific processing steps (brine injection and churning) are necessary to warrant a good penetration and diffusion of the various ingredients and additives intended for product stabilization. In our case, it might be possible that the churning conditions were not sufficient for this to happen. Furthermore, it is possible to observe that the increase in COP content might be correlated with the rapid decrease in 3,4-DHPEA content during storage (Table 3), which compromises its antioxidant activity, particularly against cholesterol. This phenomenon could be attributed to a nitration effect on the aromatic ring of phenolic compounds, which, by primarily acting as ROS scavengers, inhibit the antioxidant action of phenols against cholesterol [69].

Regarding the single COPs profile and amount detected in the present study, it is similar to the one reported for raw and cooked pork-based sausages in a previous study [47]. In general, the most abundant COP was 7-KC, followed by 7β-HC and 7α-HC, thus demonstrating that COPs mainly formed in position 7 by means of a monomolecular formation mechanism [70]. In particular, the increasing formation of 7α-HC in S3T30 is favored under mild autoxidative conditions and can be selectively promoted when phenolics with catechol/ortho-dihydroxyl units enter a redox cycle with trace metals (pro-oxidant switch), or when antioxidants preferentially partition to the aqueous phase, leaving the sterol-rich lipid microdomains less protected [71]. During oven cooking, cholesterol undergoes autoxidation and thermally induced oxidation, forming C-7 hydroperoxides that can dismutate into 7-hydroxy and 7-keto derivatives. In the other cooked ham formulations, a decrease in the 7-hydroxy derivatives coupled with an increase in 7-keto derivatives was observed, likely reflecting an equilibrium among the oxidation products during the monomolecular oxidation phase [72].

The bimolecular formation mechanism, which involves the reaction between a cholesterol molecule and a hydroperoxyl radical, was less favored in our samples; in fact, low quantities of both epoxides (α-EC 0.08–0.50 mg/kg and β-EC 0.13–1 mg/kg) were found. Low amounts of cholestanetriol (<0.54 mg/kg of meat), the main decomposition product of cholesterol epoxides, were also detected; the presence of H_2_O in an acidic environment is necessary for favoring the opening of the epoxy ring [70]. Analyzing the trends of individual COPs across the different samples, a decrease in COPs concentration was observed in sample C at T30. This phenomenon may be attributed to a shift in the equilibrium, resulting in a reduced rate of COP formation with respect to their further transformation or reaction with other matrix components (e.g., proteins), potentially leading to the formation of Schiff bases [73]. Samples S1 and S3 exhibited a bell-shaped trend, characterized by a slight inflection at the mid-point of the shelf-life study, followed by a modest increase toward the final time point, while consistently maintaining low COP levels. Cholesterol oxidation in cooked ham is influenced significantly by environmental factors such as humidity and pH, which can affect the formation of COPs. In particular, a_w_ affects the stability and reactivity of lipids and cholesterol in food. In the case of cooked ham, high a_w_ (0.98–0.99, Table 2) can facilitate oxidation reactions by providing more dissolved oxygen and a medium for reactants to interact, thus leading to increased COP formation. The high a_w_, together with a slightly acidic pH, favors the formation of some COPs, particularly CT. In the present study, CT was present in relatively low amounts (Table 6), but it is important to monitor its formation and presence because it has been associated with several relevant biological effects, including neurotoxicity, cytotoxicity, inflammatory responses, disruption of calcium homeostasis, and alteration of lipid metabolism [74]. Based on these findings, it appears that the phenolic extract did not significantly impact cholesterol oxidation in this meat product, as sample C exhibited lower COP levels compared to the PE-enriched samples. As previously explained, this could probably be due to the fact that the extract was not able to fully penetrate inside the cell membrane, where cholesterol is located. Nitrites, on the other hand, through passive diffusion, were able to enter cells and exert their antioxidant activity against cholesterol. Nitrite, in fact, can diffuse through biological membranes, but this process is significantly influenced by its chemical form. Nitrite ion (NO_2_^−^) can move across membranes, but its passive diffusion is very low due to the hydrophobic nature of the phospholipid bilayer, as it is a barrier for ionic species [75]. Studies suggest that nitrite may diffuse more readily in the form of undissociated nitrous acid (HNO_2_), especially at lower pH levels where the concentration of HNO_2_ increases [75]. This means that in acidic conditions, as in the case of cooked ham, nitrite can penetrate cell membranes more effectively. Our results highlight a clear synergistic antioxidant effect between PE and nitrites on general oxidative markers, such as protein carbonyls and TBARs, whereas this synergy appears less effective against COPs. The robust protection of carbonyls and TBARs can be attributed to the efficient radical-scavenging and lipid peroxidation-inhibiting activity of polyphenols, which is further enhanced by nitrite-mediated synergistic mechanisms [76]. In contrast, COP formation involves more complex, structure-specific oxidative pathways that are less susceptible to the general radical scavenging activity of phenols [77]. Moreover, nitrites may contribute to reactive nitrogen species formation, potentially promoting cholesterol oxidation and partially offsetting the protective effect of PE. These findings suggest that the partial failure of the PE-nitrite synergy on COPs arises from the interplay between the distinct chemical reactivity of cholesterol, specific oxidation pathways, and the dual antioxidant-pro-oxidant behavior of nitrites, highlighting the parameter-dependent nature of antioxidant interventions [76].

The extent of the cholesterol oxidation was estimated by means of the cholesterol oxidation ratio (%OR), which ranged from 0.05% to 0.34% (Table 6). Product formulation, storage time, and their interaction significantly influenced this ratio, as well as total COPs. C samples showed an increase in %OR at T15, followed by a decrease at T30. The PE-added samples, on the other hand, showed three distinct trends, which were analogous to those observed for total COPs. Nonetheless, it is important to highlight that the cholesterol oxidation rates (%OR) were notably low in all cooked ham samples, remaining below 0.5%. The %OR in cooked samples can remain low through careful selection of cooking methods, optimal storage conditions, appropriate humidity and pH levels, incorporation of natural antioxidants, and control over fatty acid composition. These strategies are essential for enhancing the safety and quality of processed meat products while minimizing health risks associated with COPs [77]. Moreover, it is important to point out that the EU currently does not possess accurate or detailed information on dietary exposure and consumption of COPs. As a result, it remains unclear whether the current intake levels in the population are safe. Therefore, it is important to limit the consumption of these substances and reduce their presence in food [78,79].

### 3.7. VOCs Profile

**Figure 1:**
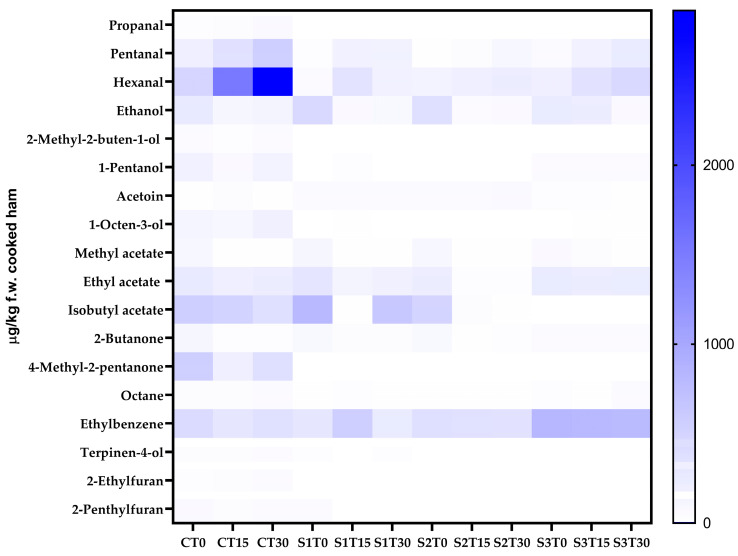
Heat map comparing the content of the volatile compounds of cooked ham samples after 0, 15, and 30 days of storage. C, Control (meat + maltodextrine + 150 mg of nitrites/kg of meat); S1, meat + 150 mg of nitrites/kg of meat + 200 mg of phenols/kg of meat; S2, meat + 35 mg of nitrites/kg of meat + 200 mg of phenols/kg of meat; S3, meat + 200 mg of phenols/kg of meat.

A complex mixture of VOCs belonging to diverse classes of compounds was identified and quantified by the SPME-GC-MS technique. As shown in Figure 1, 18 VOCs were detected in cooked ham samples, which are distributed differently depending on the type of sample analyzed. Product formulation, storage time, and their interaction significantly influenced the VOC profile. In sample C (150 mg of nitrites/kg, without PE), the most represented class was esters at T0 (27.76% of the total VOCs), while at T15 and T30, there was a relevant increase in the amount of aldehydes (54.48% and 63.94% of the total VOCs, respectively), particularly hexanal (1505.96–2863.24 µg/kg f.w. cooked ham of 4-methyl-2-pentanol equivalent). As for sample S1 (150 mg of nitrites/kg + 200 mg of PE/kg), esters were the most abundant class of VOCs at T0 (54.83% of the total VOCs); however, at T15, there was an increase in hydrocarbons (43.57% of the total VOCs), while at T30, esters became predominant again (53.85% of the total VOCs), with isobutylacetate as the most abundant compound (628.21 µg/kg f.w. cooked ham of 4-methyl-2-pentanol equivalent). Also, in sample S2 (35 mg of nitrites/kg + 200 mg of PE/kg), esters were the most represented class at T0 (43.90% of the total VOCs), while at T15 and T30, there was an increase in hydrocarbons (49.15% and 42.25% of the total VOCs, respectively), with ethylbenzene being the most abundant compound (333.51–348.38 µg/kg f.w. cooked ham of 4-methyl-2-pentanol equivalent). Finally, in sample S3 (200 mg of PE/kg, without nitrites), hydrocarbons turned out to be the most abundant class throughout the entire shelf-life (50.48%, 44.16%, and 44.13% at T0, T15, and T30, respectively), with ethylbenzene as the most abundant compound (785.91–829.64 µg/kg f.w. cooked ham of 4-methyl-2-pentanol equivalent).

The volatilome found in the present study is in line with what other authors have found in cooked ham samples [80,81]. As can be observed from Table S4, in C samples, aldehydes, especially hexanal, tend to increase during shelf-life. Aldehydes can be generated through the secondary phase of lipid oxidation (via thermal degradation and/or chemical auto-oxidation), the fermentation of sugars, and the breakdown of amino acids during the cooking process and/or storage [80,82]; in particular, during the propagation step of lipid oxidation, aldehydes are likely to accumulate, so they are considered as markers of secondary lipid oxidation, and in many food products, they are actually recognized as the main contributors to the rancid odor. Hexanal prevails as an oxidation compound, since it can originate from the oxidation of oleic, linoleic, and arachidonic acids through diverse reaction pathways. One pathway through which aldehydes are formed during the Maillard reaction is via Strecker degradation. This process involves the conversion of amino acids into volatile aroma compounds, specifically aldehydes. For instance, amino acids may be oxidized to yield aldehydes that contribute to the characteristic flavors of cooked foods [83].

Other VOCs present in cooked meat are associated with more specific oxidation routes. For instance, some aldehydes like octanal originate from oleic acid oxidation, while 2-pentylfuran derives from linolenic acid degradation. The dialkenes are by-products of linoleic acid, and 1-octen-3-ol is partially generated through arachidonic acid autoxidation [83,84].

In the present study, as can be seen from Figure 1 and Table S4, the addition of PE (200 mg/kg) to the cooked ham formulation significantly limited the development of aldehydes in samples S1, S2, and S3. It can also be observed that the synergy between nitrites and phenols ensured the best performance in terms of oxidative stability; in fact, sample S1 appears to have the lowest amount of aldehydes, followed by sample S2. Finally, the addition of PE in the nitrite-free sample (S3) also appears to be efficient in limiting the formation of aldehydes; in fact, as can be seen from Table S4, 20% lower aldehyde values were found in sample S3 T30 than in sample C T30.

In the specific case of hexanal, a well-established marker of lipid oxidation, distinct patterns were observed among the different samples. In sample C, the high baseline hexanal value indicates the presence of pre-existing hydroperoxides and/or higher processing-induced oxidation prior to T0, due to the lack of phenolic quenching (Figure 1 and Table S4). Despite the presence of antioxidant agents in brine formulation, cooking exerted a thermoxidizing effect, leading to the formation of hexanal. The strong increase in hexanal at T30, for all the samples, is therefore not related to phenol content (absent in C), but rather to thermoxidation of the PUFA-rich matrix under available O_2_ [83]. In this case, the added amount of nitrites seems to have been insufficient to effectively counteract oxidation and, consequently, hexanal formation. Finally, in S3, the high hexanal level at T30 (Figure 1 and Table S4) suggests only partial protection from phenols against oxidation, probably because, in the absence of nitrites, the synergistic effect with phenols was missing.

### 3.8. Physical Analysis

#### 3.8.1. Colorimetric Analysis

The color of the cooked ham is an important quality parameter for the consumer. The addition of phenolic compounds did not affect L* (*p* > 0.05), but it evidently influenced a* and b*, especially if evaluated as a function of the concentration of added nitrites. C and S1 showed higher a* (*p* < 0.001) and lower b* values (*p* < 0.001); formulation with lower nitrite amount (S2) or their total replacement (S3) caused a reduction in redness and an increase in yellowness (Table 7). High b* values are typical of the meat products with reduced nitrites [85].

Generally, 40–50 mg/kg of nitrites are sufficient for color development in most meat products [86]. The spray-dried PE used in this study did not contain nitrites, unlike other vegetable derivatives, where nitrites are pre-converted to accumulate a sufficient amount for adequate color development [45]. The storage did not have a significant (*p* > 0.05) effect on L*, a*, and b*, but, in general, a discoloration was noted despite the protective atmosphere packaging. This behavior could be ascribed to photochemical reactions in the presence of residual oxygen, as observed by Haile et al. [87] in sliced cooked ham packed with varying vacuum levels and different percentages of gas mixture (CO_2_-N_2_) in a modified atmosphere package (MAP). In fact, it is crucial to maintain residual oxygen between 0.1% and 0.5% to avoid light-induced oxidative discoloration [87]. Although vacuum packaging helps protecting against oxidation, transparent packages still allow light to cause discoloration [87].

To evaluate the distance between two colors, Delta E (from the color value at T0) perception is used: <1.0 is invisible to the human eye; 1–2 visible through observation; 2–10 at a glance; 11–49 colors are more similar; and 100 colors are opposite [88]. The results indicate that it was easy to perceive color change during storage (*p* < 0.001), especially for C and S3 samples.

#### 3.8.2. Image Analysis

In this study, the electronic eye was applied to discriminate between different formulations of cooked ham samples (C, S1, S2, S3) stored at three different times (0, 15, 30 days) by acquisition and subsequent processing of sample images (Alphasoft software, version 14.0).

**Figure 2:**
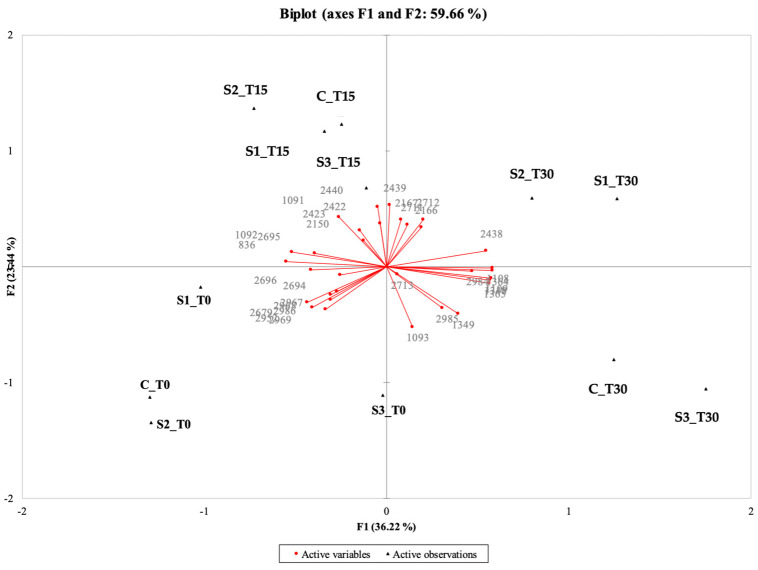
Representation of the cases and variables obtained from the principal component analysis (PCA) related to the results of the image analysis (electronic eye) for the 4 samples under examination (C, Control (meat + maltodextrin + 150 mg of nitrites/kg of meat); S1, meat + 150 mg of nitrites/kg of meat + 200 mg of phenols/kg of meat; S2, meat + 35 mg of nitrites/kg of meat + 200 mg of phenols/kg of meat; S3, meat + 200 mg of phenols/kg of meat) evaluated at all the storage times (T0, T15, and T30).

The PCA of the instrumental (electronic eye) data shows that the first two components explained 70.39% of the total variance (50.43% for PC1 and 19.96% for PC2) (Figure 2).

The samples characterized by the highest intensities of pink (CT0, S1T0, S2T0, S2T15) were placed together between the second and the third quadrant; in the same position, but more shifted towards the positive values of PC2, there were CT15 and S1T15 with intermediate pink intensities. On the other hand, samples positioned between the first and fourth quadrant are those that showed anomalous colors (CT30, S1T30, S2T30, S3T30) linked to the oxidative process, even if, in some cases, with high intensity of pink (S2T30). S3T0 and S3T15 are located far from the other samples in the PCA, which confirms their anomalous appearance; this was also detected by the trained panel (see Section 3.10.1), as these samples had a low pink intensity, were pale, and tended to be gray.

These results agree with those reported in several studies in which instrumental methods were applied to evaluate the appearance of cooked ham samples, showing a strong correlation between instrumental and sensory data [38,89,90]. In general, it was observed that the surface color value of the C sample gradually decreases with storage time. On the contrary, the PE-enriched samples with nitrite (S1, S2) revealed considerable stability of color for up to 15 days of storage. After this time, all samples evidenced a qualitative decay, with the formation of visual anomalies (discoloration and iridescence; formation of dark, yellowish, grayish areas). Similar trends were also reported by other authors [41,91], who investigated the effect of the incorporation of olive oil by-products on beef hamburgers during cold storage, confirming their effectiveness in stabilizing their sensory quality and prolonging their shelf-life.

### 3.9. PCA of Chemical and Physical Data

**Figure 3:**
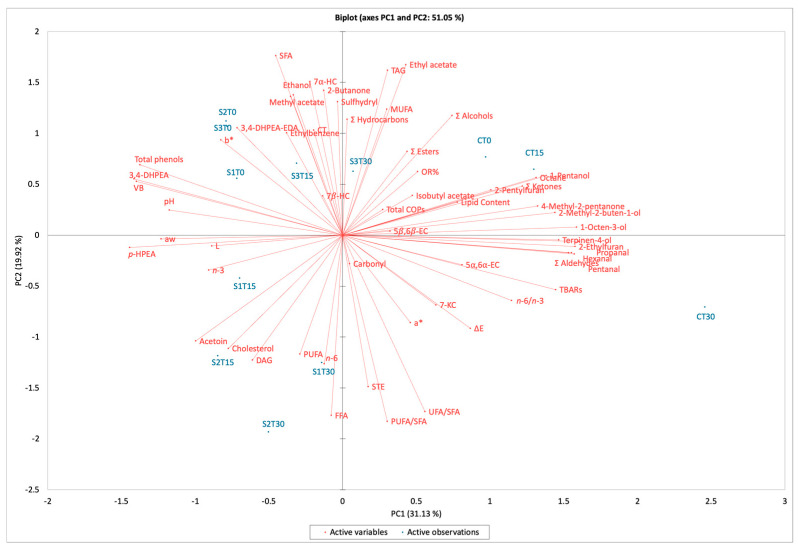
Biplot of cooked ham samples after 0, 15, and 30 days of storage. C, Control (meat + maltodextrin + 150 mg of nitrites/kg of meat); S1, meat + 150 mg of nitrites/kg of meat + 200 mg of phenols/kg of meat; S2, meat + 35 mg of nitrites/kg of meat + 200 mg of phenols/kg of meat; S3, meat + 200 mg of phenols/kg of meat. 3,4-DHPEA, hydroxytyrosol; 3,4-DHPEA-EDA, decarboxymethyl oleuropein aglycone; 5α,6α-EC, 5α,6α-epoxycholesterol; 5β,6β-EC, 5β,6β-epoxycholesterol; 7β-HC, 7β-hydroxycholesterol; 7-KC, 7-ketocholesterol; a_w_, water activity; COPs, cholesterol oxidation products; CT, cholestanetriol; DAG, diacylglycerols; FFA, free fatty acids; MAG, monoacylglycerols; MUFA, monounsaturated fatty acids; *p*-HPEA, tyrosol; OR%, cholesterol oxidation ratio; PUFA, polyunsaturated fatty acids; SFA, saturated fatty acids; STE, sterols; TAG, triacylglycerols; TBARs, thiobarbituric acid reactive substances; UFA, unsaturated fatty acids; VB, verbascoside.

To better understand which parameters were the most relevant for assessing the effects of phenolic enrichment and storage on cooked ham, the chemical composition and physical data were subjected to principal component analysis (PCA) (Figure 3). The first two components explained 51.05% of the total variance (31.13% for PC1 and 19.92% for PC2).

The biplot reveals distinct sample groups. Notably, samples S3T30 and CT15 are closely associated with higher levels of COPs and OR%. Conversely, sample CT30 shows a stronger correlation with TBARs and aldehydes, both indicators of secondary lipid oxidation. Aldehydes derive mainly from lipid oxidation (such as hexanal), but they can also originate from the Maillard reaction, which can occur to a limited extent in cooked ham due to the high A_w_ of the product; in any case, aldehydes contribute to the overall flavor profile of cooked ham. The proximity of total and single phenols in quadrants 2 and 3 for samples S1, S2, and S3, in opposite quadrants of C samples, suggests that phenolic enrichment has a consistent effect on these samples, having a protective role against protein and lipid oxidation. The slight variability of the cooked ham samples can significantly impact the PCA results, in terms of total variance, making it essential to consider biological differences when interpreting data. Overall, the PCA effectively illustrates how phenolic enrichment and storage conditions influence the chemical properties of cooked ham. The relationships identified between various compounds and sample groups provide valuable insights into potential strategies for improving oxidative stability and quality in meat products.

### 3.10. Sensory Analysis

#### 3.10.1. Descriptive Analysis

Sensory evaluation of the cooked ham samples was performed using the QDA^®^ method. The descriptor list included the following: (i) one attribute related to appearance: color intensity (degree of pinkness); (ii) three attributes perceived via orthonasal and retronasal pathways: overall aroma (intensity of the product’s aroma), spices and other flavors (intensity of added or inherent flavor notes), and smoky aroma (typical of smoked meat products); (iii) two taste-related attributes: sweetness and saltiness; and (iv) two texture-related attributes: cohesiveness (resistance to disintegration during the first 3–4 chews) and juiciness (amount of liquid released during mastication).

Mean values for these sensory descriptors (see Table 8) revealed significant differences (*p* < 0.05) in six out of eight parameters. A general decline in overall aroma intensity was observed as early as 15 days of storage (T15), with the C sample showing the most pronounced decrease. In contrast, samples S1 and S2 (both containing PE and nitrites) retained higher aroma intensity. Notably, sample S3 (PE-enriched, nitrite-free) exhibited the lowest overall aroma intensity from the beginning (T0) and throughout storage.

Smoky aroma significantly declined from T0 to T30 in samples C and S3, while S1 and S2 maintained their smoky notes over time. At T0, the salty taste was significantly more intense in the control sample (C) and remained stable during storage.

With regard to texture attributes, the enrichment with phenolic compounds did not affect the cohesiveness of the cooked ham samples but contributed to maintaining this attribute stable over time. In contrast, samples C and S3 exhibited a progressive decrease in cohesiveness from T0 to T15 and T30. As for juiciness, a reduction was observed over time in sample C, showing the highest value at T0. In samples S1 and S2, juiciness remained relatively constant, with no significant changes during storage. Interestingly, an increase in juiciness was noted for sample S3, likely linked to product deterioration in the absence of nitrites.

Regarding color intensity, particularly the pink hue, the sensory panel reported a decline in samples C and S1 from T0 to T15 and T30. On the other hand, samples S2 and S3 did not show significant changes over time in this visual attribute. In the case of S2, this stability could be attributed to the combined presence of phenolic extract and nitrites, which are known to contribute to color retention. In sample S3, however, the consistently lower values were due to the absence of nitrites in the formulation. This absence prevented the formation of nitrosyl hemochrome, the compound responsible for the characteristic pink color in cooked cured meats [4]. Furthermore, an excess of antioxidants, such as polyphenols, may interfere with the formation of this pigment by reacting with sodium nitrite to produce nitrogen gases (N_2_ or N_2_O), thereby limiting nitric oxide availability and its reaction with myoglobin [92].

In addition to rating the intensity of sensory descriptors using a linear scale, panelists were also asked to identify the presence of off-flavors or negative sensory notes. Olfactory anomalies with fermented or lactic characteristics were detected in samples C, S1, and S3 at T1 and T15, while for sample S2, such anomalies appeared only at T30. These observations are consistent with literature reports on the sensory evolution of sliced cooked ham stored in modified atmosphere, where discoloration and off-flavors (such as sour or rotten notes) are considered key indicators of microbial spoilage [93,94].

Gustatory defects, including sour and rancid notes, emerged at T15 and T30 in sample C and only at T30 in the PE-enriched samples (S1, S2, and S3). In the case of S3, sensory evaluators already perceived anomalous notes resembling cooked meat at T0, becoming more pronounced over time. Additionally, abnormal surface coloration, including dark, yellowish, or grayish areas, was observed in all samples except for CT0, S2T0, and S2T15.

The presence of PE, even in combination with reduced nitrite levels, proved to be effective in limiting the formation of sensory defects. Among the tested formulations, sample S2 demonstrated the highest capacity to delay the onset of rancid and off-flavor notes, confirming findings from previous studies in which phenol-rich extracts from olive mill wastewaters were used to improve the quality and shelf-life of meat products [41,95].

**Figure 4:**
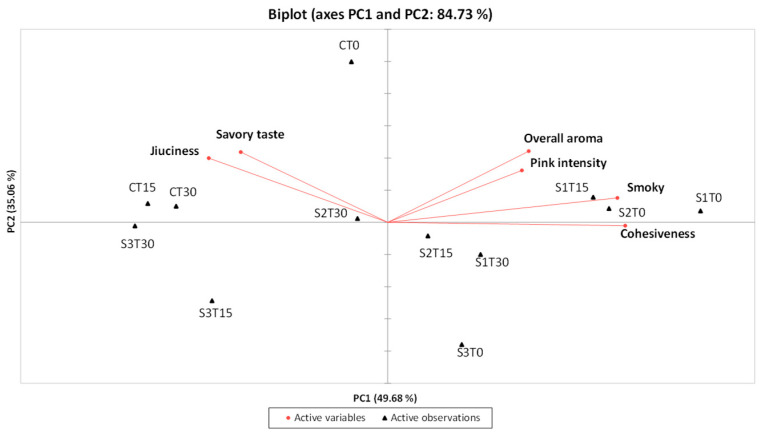
PCA related to the results of the QDA^®^ for the 4 samples under examination (C, Control (meat + maltodextrin + 150 mg of nitrites/kg of meat); S1, meat + 150 mg of nitrites/kg of meat + 200 mg of phenols/kg of meat; S2, meat + 35 mg of nitrites/kg of meat + 200 mg of phenols/kg of meat; S3, meat + 200 mg of phenols/kg of meat)) evaluated at all the storage times (T0, T15, and T30).

To further explore the relationships between samples and sensory attributes, a PCA was performed using the descriptors that showed significant differences according to ANOVA. The projection of the samples onto the first two components accounted for 84.73% of the total variance, with PC1 and PC2 explaining 49.68% and 35.06%, respectively (Figure 4).

Samples enriched with phenolic extract and nitrites, specifically S1T0, S1T15, and S2T0, were positioned in the first quadrant of the PCA plot, distinguished by a strong overall aroma, pronounced smoky notes, and high pink intensity. In contrast, the control samples (CT0, CT15, and CT30), along with S2T30, were located in the second quadrant and were characterized by a marked juiciness (particularly CT15 and CT30) and a pronounced savory taste. Samples from the S3 group stored for 15 and 30 days (S3T15 and S3T30) were placed in the third quadrant, notable for their low pink color intensity and reduced perception of aroma-related attributes, both orthonasal and retronasal (overall aroma and smoky notes). Finally, in the fourth quadrant, sample S1T30 showed a decrease in overall aroma compared to S1T15, while sample S2T15, which was very similar to S2T30, exhibited slightly lower juiciness; on the other hand, sample S3T0 was characterized by a more intense smoky note and lower juiciness compared to S3T15 and S3T30.

#### 3.10.2. Discriminant Test

A triangle test was conducted to assess whether significant sensory differences existed between the treated samples (S1, S2, and S3) and the control sample (C) at various storage times (0, 15, and 30 days). The test was carried out over multiple sessions to ensure that all possible combinations of samples were presented to the panelists for comparison (Table 9).

Sessions 1 to 6 aimed to compare all samples (C, S1, S2, S3) at time 0 (T0). The results obtained from 44 subjects revealed significant differences between all sample pairs, except between S1T0 and S2T0. Specifically, S1T0 was distinguished from CT0 by a more intense pink color and a firmer, less juicy texture. CT0 differed from S2T0 due to its lower overall aroma and pink intensity, but greater tenderness and juiciness. CT0 was also distinguished from S3, which showed lower color intensity, overall aroma, and juiciness. The S3 sample, being nitrite-free, was clearly distinguishable from the others and characterized by a paler, grayish color, lower aroma intensity, and reduced juiciness.

Sessions 7 to 12 evaluated samples after 15 days of storage at 4 °C (T15). All samples were significantly different, even though lower significance levels were observed in some comparisons (e.g., CT15 vs. S2T15 and S1T15 vs. S2T15). Both S1T15 and S2T15 were perceived as darker and drier than CT15. The S3T15 sample was characterized by a distinctly gray color and lower overall aroma compared to C, while also being juicier than the other nitrite- and PE-enriched samples.

In sessions 13 to 18, samples were compared after 30 days of storage (T30). The subjects (n = 29) correctly identified the different samples in each case, even though lower statistical significance was noted in one comparison (CT30 vs. S2T30). Sample C differed from S1 and S2 in having a less intense pink color, greater firmness and juiciness, and the presence of off-flavors described as rancid or fermented. The S3 sample remained clearly distinguishable at T30 due to its anomalous gray color, cooked meat aroma, and sensory notes perceived as more acidic, juicy, and fermented. Significant differences were also observed between S1T30 and S2T30, with S2 displaying a higher pink intensity and juiciness. In conclusion, participants were able to discriminate between the tested samples, supporting findings from the trained sensory panel. The combination of phenolic extract and nitrites contributed to greater color stability, notably reducing pink color loss over time. This stabilizing effect was especially evident in the S2 sample (PE-enriched with a reduced nitrite dose), confirming previous studies that demonstrated the effectiveness of olive-derived phenolic compounds as natural additives in meat products [41,63]. Notably, the addition of phenols did not result in perceivable olfactory or gustatory alterations. This aspect is critical for consumer acceptance, as phenol-rich extracts, especially from olive mill wastewaters, may impart sensory notes resembling their origin (e.g., bitterness or pungency), depending on concentration, product type, and formulation [20].

## 4. Conclusions

This study highlights the potential of using brine enriched with phenolic extracts from olive vegetation water (OVW) to enhance the oxidative stability and overall quality of cooked ham during storage. Although there was a gradual reduction in phenolic content over time, the remaining phenols contributed to the inhibition of lipid and protein oxidation. In particular, when the OVW-enriched brine was combined with reduced nitrite levels, it effectively limited the formation of secondary oxidation products such as TBARs, volatile aldehydes, and cholesterol oxides. Moreover, sensory analysis confirmed that the inclusion of the extract did not negatively affect the organoleptic properties of the product and helped maintaining color stability during storage. In conclusion, these findings support the feasibility of using OVW-derived phenolic compounds in brine as a natural alternative for partial replacement of nitrites in cooked ham formulations. This is significant evidence in view of the reduction in the maximum usable concentration of nitrite in cooked meat products as foreseen by European legislation. This approach addresses growing consumer demand for cleaner-label meat products while contributing to the sustainable valorization of olive oil industry by-products within a circular economy framework. Further research is needed to optimize formulation strategies and evaluate the microbial stability of nitrite-reduced cooked ham (considering also the spore-forming bacteria), in order to ensure both safety and quality over extended storage periods.

## Figures and Tables

**Table 1:** Cooking program for cooked ham.

Oven Temperature (°C)	Core Temperature Target (°C)
40	20
55	40
73	69

**Table 2:** Proximate composition, water activity, and pH of cooked hams after 0, 15, and 30 days of storage. C, Control (meat + maltodextrin + 150 mg of nitrites/kg of meat); S1, meat + 150 mg of nitrites/kg of meat + 200 mg of phenols/kg of meat; S2, meat + 35 mg of nitrites/kg of meat + 200 mg of phenols/kg of meat; S3, meat + 200 mg of phenols/kg of meat.

										SEM	*P*		
		NaCl									Form	St	Form*St
Storage Time (days)		C		S1		S2		S3		0.08	*NS*	*NS*	*NS*
	0	1.94	b, B	2.05	a, A	1.82	b, B	2.16	c, A				
	15	2.51	a, A	1.92	ab, B	1.95	a, B	2.41	a, A				
	30	1.94	b, B	1.88	b, B	1.57	c, C	2.25	b, A				
		Protein											
		C		S1		S2		S3		0.45	*NS*	*NS*	*NS*
	0	27.63	a, A	27.12	a, A	25.14	a, C	26.32	a, B				
	15	21.85	c, C	25.77	b, A	23.49	b, B	25.89	b, A				
	30	25.99	b, A	25.72	b, A	25.25	a, A	24.92	b, B				
		Moisture											
		C		S1		S2		S3		0.37	*NS*	*NS*	*NS*
	0	64.30	a, B	66.08	a, A	64.42	a, B	63.05	b, C				
	15	64.16	a, B	66.04	a, A	63.08	b, C	63.57	b, C				
	30	64.32	a, A	62.41	b, B	61.93	c, B	64.47	a, A				
		Fat											
		C		S1		S2		S3		0.64	*	*NS*	*NS*
	0	8.07	c, B	6.80	c, C	10.45	c, A	10.63	a, A				
	15	13.99	a, A	8.19	b, C	13.43	a, A	10.54	a, B				
	30	9.70	b, D	11.87	a, B	12.82	b, A	10.61	a, C				
		A_w_											
		C		S1		S2		S3		0.00	***	*	***
	0	0.985	*NS*	0.995	*NS*	0.984	*NS*	0.983	*NS*				
	15	0.982	*NS*	0.985	*NS*	0.985	*NS*	0.983	*NS*				
	30	0.980	*NS*	0.983	*NS*	0.984	*NS*	0.984	*NS*				
		pH											
		C		S1		S2		S3		0.05	***	***	***
	0	5.95	a, B	6.43	a, A	6.50	a, A	5.97	b, B				
	15	5.96	a, B	6.42	a, A	6.24	a, A	6.04	a, AB				
	30	5.73	a, B	6.08	a, A	5.98	b, B	5.88	b, B				

Results are expressed as means and standard error of the mean (SEM) of 3 independent replicates. a–c indicates significant differences (Tukey’s test; *p* ≤ 0.05) within the same treatment during the shelf-life. A–D indicates significant differences (Tukey’s test; *p* ≤ 0.05) among treatments, * *p* < 0.05, *** *p* < 0.001. Form, formulation; *NS*, non-significant; St, storage.

**Table 3:** Evolution of phenolic compounds (mg/kg of cooked ham) of cooked hams after 0, 15, and 30 days of storage. C, Control (meat + maltodextrin + 150 mg of nitrites/kg of meat); S1, meat + 150 mg of nitrites/kg of meat + 200 mg of phenols/kg of meat; S2, meat + 35 mg of nitrites/kg of meat + 200 mg of phenols/kg of meat; S3, meat + 200 mg of phenols/kg of meat.

									SEM	*P*		
	3,4-DHPEA									Form	St	Form*St
Storage Time (days)		C	S1		S2		S3		5.90	***	***	***
	0	-	63.52	a, B	80.62	a, A	69.09	a, B				
	15	-	60.12	b, B	48.40	b, C	66.83	a, A				
	30	-	38.15	c, A	24.86	c, C	31.19	b, B				
	*p*-HPEA											
		C	S1		S2		S3		0.64	***	*	*
	0	-	6.15	a, C	6.59	b, B	6.90	c, A				
	15	-	6.20	a, C	7.29	a, B	8.35	a, A				
	30	-	6.14	a, B	6.34	b, B	8.00	b, A				
	VB											
		C	S1		S2		S3		2.39	***	***	***
	0	-	38.01	a, A	31.37	a, B	38.41	a, A				
	15	-	30.11	b, A	18.07	b, B	32.91	b, A				
	30	-	23.06	c, A	15.96	c, B	23.66	c, A				
	3,4-DHPEA-EDA											
		C	S1		S2		S3		2.87	***	***	***
	0	-	33.83	a, A	21.56	a, B	36.47	a, A				
	15	-	*n.d.*	b	*n.d.*	b	*n.d.*	b				
	30	-	*n.d.*	b	*n.d.*	b	*n.d.*	b				
	Total phenols											
		C	S1		S2		S3		11.17	***	***	***
	0	-	141.51	a, B	140.13	a, B	150.86	a, A				
	15	-	96.42	b, B	73.76	b, C	108.08	b, A				
	30	-	67.35	c, A	47.16	c, B	62.84	c, A				

Results are expressed as means and standard error of the mean (SEM) of 3 independent replicates. a–c indicates significant differences (Tukey’s test; *p* ≤ 0.05) within the same treatment during the shelf-life. A–C indicates significant differences (Tukey’s test; *p* ≤ 0.05) among treatments, * *p* < 0.5, *** *p* < 0.001. 3,4-DHPEA, hydroxytyrosol; Form, formulation; *p*-HPEA, tyrosol; St, storage; VB, verbascoside; 3,4-DHPEA-EDA, oleacein; *n.d.*: not detected.

**Table 4:** Profile of main lipid classes (% of total lipids) of cooked hams after 0, 15, and 30 days of storage. C, Control (meat + maltodextrin + 150 mg of nitrites/kg of meat); S1, meat + 150 mg of nitrites/kg of meat + 200 mg of phenols/kg of meat; S2, meat + 35 mg of nitrites/kg of meat + 200 mg of phenols/kg of meat; S3, meat + 200 mg of phenols/kg of meat.

										SEM	*P*		
	FFA										Form	St	Form*St
Storage Time (days)		C		S1		S2		S3		0.11	***	***	***
	0	1.08	ab, A	0.82	b, C	0.96	c, B	0.90	a, B				
	15	0.88	b, C	1.15	a, B	1.90	b, A	0.83	b, C				
	30	1.57	a, B	1.10	a, C	2.47	a, A	0.87	b, D				
	STE												
		C		S1		S2		S3		0.07	***	***	***
	0	1.12	a, A	0.89	b, C	0.61	b, B	0.95	a, B				
	15	0.50	b, C	0.86	b, B	1.03	ab, A	0.50	b, C				
	30	1.19	a, AB	1.04	a, B	1.42	a, A	0.57	b, C				
	DAG												
		C		S1		S2		S3		0.15	***	***	***
	0	5.15	a, A	5.32	ab, A	4.93	b, B	4.92	a, B				
	15	4.68	b, B	5.50	a, A	4.90	b, B	4.24	ab, B				
	30	5.12	a, A	5.46	a, A	5.51	a, A	4.56	ab, B				
	TAG												
		C		S1		S2		S3		0.29	***	***	***
	0	92.82	b, B	93.02	a, B	93.45	a, A	93.46	ab, A				
	15	93.71	a, A	92.47	b, B	92.72	b, B	93.95	ab, A				
	30	92.40	b, B	91.84	c, C	91.40	c, C	94.00	ab, A				

Results are expressed as means and standard error of the mean (SEM) of 3 independent replicates. a–c indicates significant differences (Tukey’s test; *p* ≤ 0.05) within the same treatment during the shelf-life. A–D indicates significant differences (Tukey’s test; *p* ≤ 0.05) among treatments, *** *p* < 0.001. DAG, diacylglycerols; FFA, free fatty acids; Form, formulation; STE, sterols; St, storage; TAG, triacylglycerols.

**Table 5:** Fatty acid classes (% of total fatty acids) and their ratios in cooked hams after 0, 15, and 30 days of storage. C, Control (meat + maltodextrin + 150 mg of nitrites/kg of meat); S1, meat + 150 mg of nitrites/kg of meat + 200 mg of phenols/kg of meat; S2, meat + 35 mg of nitrites/kg of meat + 200 mg of phenols/kg of meat; S3, meat + 200 mg of phenols/kg of meat.

										SEM	*P*		
	SFA										Form	St	Form*St
Storage Time (days)		C		S1		S2		S3		0.67	*NS*	***	*NS*
	0	14.83	b, B	13.63	b, C	14.63	a, B	15.62	a, A				
	15	15.91	a, A	15.00	a, A	11.37	b, C	14.42	b, B				
	30	14.23	b, A	12.51	b, B	13.58	b, A	13.77	b, A				
	MUFA												
		C		S1		S2		S3		0.38	***	*	***
	0	70.92	c, B	71.38	ab, B	74.28	a, A	73.09	a, A				
	15	72.94	a, A	70.95	a, B	75.47	b, B	73.35	b, A				
	30	73.53	ab, A	71.13	b, B	72.31	b, AB	74.42	b, A				
	PUFA												
		C		S1		S2		S3		0.38	***	*NS*	**
	0	14.26	a, B	15.00	b, A	11.10	b, C	11.30	b, C				
	15	11.17	c, D	14.06	b, A	13.16	a, B	12.24	a, C				
	30	12.25	b, D	16.37	a, A	14.13	a, B	11.82	b, C				
	*n*-3												
		C		S1		S2		S3		0.08	*	**	**
	0	2.25	a, AB	2.41	a, A	1.64	b, C	2.05	a, B				
	15	1.44	b, B	2.22	b, A	2.01	a, AB	1.65	b, B				
	30	1.41	b, B	2.03	b, A	2.05	a, A	1.58	b, B				
	*n*-6												
		C		S1		S2		S3		0.33	***	**	**
	0	12.01	a, A	12.59	b, A	9.46	c, B	9.26	b, B				
	15	9.73	c, B	11.84	c, A	11.15	b, A	10.60	a, AB				
	30	10.84	b, C	14.34	a, A	12.08	a, B	10.25	ab, C				
	*n*-6/*n*-3												
		C		S1		S2		S3		0.18	*	***	**
	0	5.35	c, B	5.23	b, B	5.77	a, A	4.55	b, C				
	15	6.76	b, A	5.38	b, B	5.55	a, B	6.45	a, A				
	30	7.72	a, A	7.06	a, A	5.89	a, C	6.51	a, B				
	PUFA/SFA												
		C		S1		S2		S3		0.10	*NS*	**	*NS*
	0	0.96	a, B	1.10	b, A	0.76	c, C	0.72	b, C				
	15	0.70	b, C	0.95	c, B	1.21	a, A	0.85	a, B				
	30	0.93	a, C	1.31	a, A	1.04	b, B	0.86	a, C				
	UFA/SFA												
		C		S1		S2		S3		0.61	*NS*	**	*NS*
	0	5.75	b, B	6.34	b, A	5.84	c, B	5.40	b, B				
	15	5.29	b, B	5.67	c, B	7.80	a, A	5.94	b, B				
	30	6.03	a, B	6.99	a, A	6.36	b, B	6.27	a, B				

Results are expressed as means and standard error of the mean (SEM) of 3 independent replicates. a–c indicates significant differences (Tukey’s test; *p* ≤ 0.05) within the same treatment during the shelf-life. A–D indicates significant differences (Tukey’s test; *p* ≤ 0.05) among treatments, * *p* < 0.05, ** *p* < 0.01, *** *p* < 0.001. Form, formulation; MUFA, monounsaturated fatty acids; *NS*, non-significant; PUFA, polyunsaturated fatty acids; SFA, saturated fatty acids; St, storage; UFA, unsaturated fatty acids.

**Table 6:** Evolution of sulfhydryl (nmol SH/mg protein), carbonyl (nmol DNPH/mg protein), TBARs (mg MDA/kg of meat), total cholesterol (mg/kg of meat), single and total COPs (mg/kg of meat), and OR (%) of cooked hams after 0, 15, and 30 days of storage. C, Control (meat + maltodextrin + 150 mg of nitrites/kg of meat); S1, meat + 150 mg of nitrites/kg of meat + 200 mg of phenols/kg of meat; S2, meat + 35 mg of nitrites/kg of meat + 200 mg of phenols/kg of meat; S3, meat + 200 mg of phenols/kg of meat.

										SEM	*P*		
	Sulfhydryl										Form	St	Form*St
Storage Time (days)		C		S1		S2		S3		0.33	***	***	***
	0	12.71	a, A	8.62	b, B	8.90	a, B	13.02	a, A				
	15	12.24	a, A	10.32	a, B	6.02	b, D	8.14	b, C				
	30	5.39	c, C	7.89	c, A	6.28	b, B	6.77	c, B				
	Carbonyl												
		C		S1		S2		S3		0.54	***	***	***
	0	3.79	a, A	1.90	b, B	1.92	b, B	0.59	c, C				
	15	2.49	b, C	2.39	a, C	6.94	a, A	3.22	a, B				
	30	2.50	b, AB	1.15	c, B	1.29	b, B	2.81	b, A				
	TBARs												
		C		S1		S2		S3		0.17	***	***	***
	0	1.19	c, A	1.17	a, A	0.98	b, B	0.64	c, C				
	15	2.04	b, A	1.00	b, B	1.20	b, B	1.01	b, B				
	30	3.83	a, A	1.02	b, C	1.45	a, B	1.28	a, B				
	Cholesterol												
		C		S1		S2		S3		57.63	**	*NS*	*NS*
	0	1244.39	a, BC	1163.98	b, C	1746.82	a, A	1381.31	a, B				
	15	1190.40	b, D	1531.07	b, B	1682.34	b, A	1228.57	b, C				
	30	1272.46	a, D	1872.59	a, A	1608.03	b, B	1355.78	a, C				
	7α-HC												
		C		S1		S2		S3		0.07	*NS*	**	**
	0	0.43	a, C	0.67	a, B	0.95	a, A	0.33	b, D				
	15	0.47	a, A	0.05	b, C	0.15	b, B	0.14	b, B				
	30	0.07	b, B	0.06	b, B	0.04	b, B	0.77	a, A				
	7β-HC												
		C		S1		S2		S3		0.11	***	***	***
	0	0.78	a, B	0.75	b, B	0.73	a, B	0.83	b, A				
	15	0.78	a, A	0.49	b, B	0.24	b, B	0.54	b, B				
	30	0.17	b, C	2.05	a, A	0.12	b, C	1.63	a, B				
	5β,6β-EC												
		C		S1		S2		S3		0.10	**	***	***
	0	0.23	b, B	0.26	b, B	0.56	a, A	0.23	b, B				
	15	0.99	a, A	0.40	b, C	0.13	b, D	0.57	b, B				
	30	0.46	b, B	1.53	a, A	0.22	b, C	1.35	a, A				
	5α,6α-EC												
		C		S1		S2		S3		0.04	*	***	*
	0	0.08	b	0.05	b	0.06	b	0.04	b				
	15	0.45	a, A	0.16	b, B	0.05	b, C	0.17	b, B				
	30	0.29	b, B	0.49	a, A	0.11	a, C	0.43	a, A				
	CT												
		C		S1		S2		S3		0.03	*	*	*
	0	0.13	b, B	0.12	a, B	0.54	a, A	0.11	b, B				
	15	0.20	a, A	0.05	b, B	0.07	b, B	0.09	b, B				
	30	0.08	b, B	0.12	a, A	0.07	b, B	0.18	a, A				
	7-KC												
		C		S1		S2		S3		0.11	***	***	***
	0	0.72	b, A	1.30	a, C	0.62	b, B	0.52	b, C				
	15	0.89	b, A	0.34	b, C	0.24	b, C	0.48	b, B				
	30	1.30	a, C	2.01	a, A	1.17	a, D	1.62	a, B				
	Total COPs												
		C		S1		S2		S3		0.34	***	***	***
	0	2.36	b, B	2.46	b, B	3.46	a, A	2.04	b, C				
	15	3.78	a, A	1.50	b, C	0.88	b, D	2.01	b, B				
	30	2.37	b, C	6.27	a, A	1.73	b, D	5.99	a, B				
	OR												
		C		S1		S2		S3		0.02	***	***	***
	0	0.19	b, A	0.21	b, A	0.20	a, A	0.15	b, B				
	15	0.32	a, A	0.10	b, B	0.05	b, C	0.16	b, B				
	30	0.19	b, B	0.34	a, A	0.11	b, C	0.44	a, A				

Results are expressed as means and standard error of the mean (SEM) of 3 independent replicates. a–c indicates significant differences (Tukey’s test; *p* ≤ 0.05) within the same treatment during the shelf-life. A–D indicates significant differences (Tukey’s test; *p* ≤ 0.05) among treatments, * *p* < 0.05, ** *p* < 0.01, *** *p* < 0.001. 5α,6α-EC, 5α,6α-epoxycholesterol; 5β,6β-EC, 5β,6β-epoxycholesterol; 7β-HC, 7β-hydroxycholesterol; 7-KC, 7-ketocholesterol; CT, cholestanetriol; Form, formulation; *NS*, non-significant; OR, oxidation ratio; St, storage; TBARs, thiobarbituric acid reactive substances.

**Table 7:** Color analysis of cooked ham samples after 0, 15, and 30 days of storage. C, Control (meat + maltodextrin + 150 mg of nitrites/kg of meat); S1, meat + 150 mg of nitrites/kg of meat + 200 mg of phenols/kg of meat; S2, meat + 35 mg of nitrites/kg of meat + 200 mg of phenols/kg of meat; S3, meat + 200 mg of phenols/kg of meat.

										SEM	*P*		
	L										Form	St	Form*St
Storage Time (days)		C		S1		S2		S3		0.52	*NS*	*NS*	*NS*
	0	64.96	b, B	67.32	a, A	67.57	b, A	64.70	b, B				
	15	66.84	a, B	65.58	b, B	70.49	a, A	64.29	b, C				
	30	62.57	c, B	65.59	b, AB	65.50	c, AB	66.73	a, A				
	a*												
		C		S1		S2		S3		0.56	***	*NS*	*NS*
	0	9.70	a, A	9.25	a, AB	8.41	a, B	4.16	a, C				
	15	9.35	b, A	9.36	a, A	8.17	a, B	2.81	b, C				
	30	9.63	a, A	9.37	a, A	8.74	a, B	2.38	b, C				
	b*												
		C		S1		S2		S3		0.42	***	*NS*	*NS*
	0	10.38	a, C	10.04	a, C	12.53	a, B	14.39	a, A				
	15	8.26	b, C	8.91	b, C	11.29	b, B	13.55	b, A				
	30	8.98	b, C	10.20	a, B	10.22	c, B	13.03	b, A				
	∆E												
		C		S1		S2		S3		0.33	***	***	***
	0	-	c, A	-	c, A	-	c, A	-	c, A				
	15	2.63	b, B	1.13	b, C	1.93	b, C	3.92	a, A				
	30	4.72	a, A	2.75	a, C	2.65	a, C	3.05	b, B				

Results as reported as means and standard error of the mean (SEM) of 3 independent replicates. a–c indicates significant differences (Tukey’s test; *p* ≤ 0.05) within the same sample during the shelf-life. A–C indicate significant differences (Tukey’s test; *p* ≤ 0.05) among treatments, *** *p* < 0.001. Form, formulation; *NS*, non-significant; St, storage.

**Table 8:** Mean values (three replicates) of the intensity of the attributes evaluated by QDA^®^ relative to all samples (C, Control (meat + maltodextrin + 150 mg of nitrites/kg of meat); S1, meat + 150 mg of nitrites/kg of meat + 200 mg of phenols/kg of meat; S2, meat + 35 mg of nitrites/kg of meat + 200 mg of phenols/kg of meat; S3, meat + 200 mg of phenols/kg of meat) evaluated at all the storage times (T0, T15, and T30). Values were expressed on a scale from 0 to 100 (0 indicates the absence of perception of the attribute, 100 the maximum perception of the attribute).

										Pr > F (Model)	Significant
	Overall aroma										
Storage Time (days)		C		S1		S2		S3		<0.0001	Yes
	0	5.9	ab	5.7	abc	5.6	abcd	4.0	e		
	15	4.7	de	6.0	a	4.8	cde	4.0	e		
	30	5.0	bcde	4.9	cde	4.9	cde	4.7	de		
	Spices and flavors										
		C		S1		S2		S3		0.294	No
	0	3.1	a	2.7	abc	2.6	abc	2.0	abc		
	15	2.2	abc	2.5	abc	2.4	abc	1.5	c		
	30	1.7	bc	2.7	ab	2.0	abc	2.0	abc		
	Smoky										
		C		S1		S2		S3		0.007	Yes
	0	2.3	ab	2.3	ab	2.5	a	2.0	abc		
	15	1.0	c	2.4	ab	1.4	abc	1.0	c		
	30	0.9	c	1.7	abc	1.4	bc	1.0	c		
	Olfactory anomalies										
		C		S1		S2		S3			
	0	/		/		/		Cooked meat (roast)			
	15	Fermented/ lactic		Fermented/ lactic		/		Cooked meat (roast); Fermented/ lactic			
	30	Fermented/ lactic		Fermented/ lactic		Fermented/lactic		Cooked meat (roast); Fermented/ lactic			
	Sweet										
		C		S1		S2		S3		0.494	No
	0	4.3	ab	4.5	ab	4.5	ab	4.6	a		
	15	4.3	ab	4.0	ab	4.4	ab	3.7	b		
	30	4.3	ab	4.1	b	4.1	ab	3.6	ab		
	Salty										
		C		S1		S2		S3		0.009	Yes
	0	4.3	a	2.6	bc	2.5	bc	2.3	c		
	15	3.4	ab	2.8	bc	2.7	bc	2.8	bc		
	30	3.3	abc	2.7	bc	2.7	bc	3.4	ab		
	Taste anomalies										
		C		S1		S2		S3			
	0	/		/		/		Cooked meat (roast)			
	15	Sourish/Rancid		/		/		Cooked meat (roast)			
	30	Sourish/Rancid		Sourish/Rancid		Sourish/Rancid		Cooked meat (roast); Sourish/Rancid			
	Cohesiveness										
		C		S1		S2		S3		0.0003	Yes
	0	4.9	bc	6.1	a	4.9	abc	5.2	ab		
	15	3.6	d	5.2	ab	4.6	bcd	4.2	bcd		
	30	3.7	d	5.3	ab	4.2	bcd	3.9	cd		
	Juiciness										
		C		S1		S2		S3		<0.0001	Yes
	0	3.9	a	1.5	ef	1.7	def	1.2	f		
	15	3.0	b	1.8	def	1.8	def	2.2	cde		
	30	2.8	bc	1.8	def	2.5	bcd	3.0	bc		
	Pink intensity										
		C		S1		S2		S3		<0.0001	Yes
	0	5.2	a	6.0	a	5.5	a	0.3	c		
	15	3.3	b	3.6	b	6.0	a	0.6	c		
	30	3.0	b	3.6	b	6.0	a	0.6	c		
	Visual anomalies										
		C		S1		S2		S3			
	0	/		Abnormal coloring (yellowish areas)		/		Abnormal coloring (gray)			
	15	Abnormal coloring (iridescent and/or dark areas)		Abnormal coloring (grayish areas)		/		Abnormal coloring (gray)			
	30	Abnormal coloring (grayish areas)		Abnormal coloring (grayish areas)		Abnormal coloring (grayish areas)		Abnormal coloring (gray)			

Different letters (a–f) indicate significantly different values from each other (multiple comparison test, Fisher LDS with *p* < 0.05). Pr, probability.

**Table 9:** Sessions, tested samples, number of untrained subjects, correct responses, and significance levels for the triangle test performed on cooked ham samples (C, Control (meat + maltodextrin + 150 mg of nitrites/kg of meat); S1, meat + 150 mg of nitrites/kg of meat + 200 mg of phenols/kg of meat; S2, meat + 35 mg of nitrites/kg of meat + 200 mg of phenols/kg of meat; S3, meat + 200 mg of phenols/kg of meat)) evaluated at all the storage times (T0, T15, and T30).

Session Number	Judges Number	Compared Samples	Corrected Answers	Significance
1	44	CT0 vs. S1T0	28	0.001
2	44	CT0 vs. S2T0	29	0.001
3	44	CT0 vs. S3T0	44	0.001
4	44	S1T0 vs. S2T0	14	*NS*
5	44	S1T0 vs. S3T0	39	0.001
6	44	S2T0 vs. S3T0	43	0.001
7	40	CT15 vs. S1T15	28	0.001
8	40	CT15 vs. S2T15	17	0.1
9	40	CT15 vs. S3T15	36	0.001
10	40	S1T15 vs. S2T15	19	0.05
11	40	S1T15 vs. S3T15	26	0.001
12	40	S2T15 vs. S3T15	32	0.001
13	29	CT30 vs. S1T30	17	0.01
14	29	CT30 vs. S2T30	23	0.001
15	29	CT30 vs. S3T30	27	0.001
16	29	S1T30 vs. S2T30	20	0.001
17	29	S1T30 vs. S3T30	24	0.001
18	29	S2T30 vs. S3T30	20	0.001

The significance is expressed in terms of α-risk level. *NS* indicates no significant perceptible difference between samples was found.

## Data Availability

Data will be made available upon request.

## Supplementary Materials

The following supporting information can be downloaded at: https://www.mdpi.com/article/10.3390/antiox14091124/s1, Table S1: Cooking weight loss and technical yield; Table S2: Microbial targets (CFU/g) of cooked ham samples after 0, 15, and 30 days of storage; Table S3: Fatty acid profile of cooked ham samples after 0, 15, and 30 days of storage; Table S4: Volatile organic compounds (expressed as µg/kg f.w. cooked ham of 4-methyl-2-pentanol equivalent) profile of cooked ham samples after 0, 15, and 30 days of storage. References [24,96,97,98] are cited in the supplementary materials.
