# Supplementary material for: Brine Enriched with Olive Wastewater Phenols: A Green Strategy to Reduce Nitrites in Cooked Ham
Source: Antioxidants (Basel). 2025 Sep 17;14(9):1124. doi: 10.3390/antiox14091124 (PMC12466680; doi:10.3390/antiox14091124)
Supplement: Supplementary file 1 [file antioxidants-14-01124-s001.zip › antioxidants-3792567-supplementary.pdf]

antioxidants

MDPI

Article

Brine Enriched with Olive Wastewater Phenols: A Green Strategy to Reduce Nitrites in Cooked Ham

Dario Mercatante [1], Stefania Balzan [2], Sonia Esposto [3], Sara Barbieri [1], Federico Fontana [2], Luca Fasolato [2], Vincenzo De Rosa [1], Maurizio Servili [3], Agnese Taticchi [3,*], Enrico Novelli [2] and Maria Teresa Rodriguez-Estrada [1,4]

[1] Department of Agricultural and Food Sciences, Alma Mater Studiorum—University of Bologna, 40127 Bologna, BO, Italy; dario.mercatante2@unibo.it (D.M.); sara.barbieri@unibo.it (S.B.); vincenzodr91@outlook.it (V.D.R.); maria.rodriguez@unibo.it (M.T.R.-E.)
[2] Department of Comparative Biomedicine and Food Science, University of Padova, 35020 Legnaro, PD, Italy; stefania.balzan@unipd.it (S.B.); federico.fontana@unipd.it (F.F.); luca.fasolato@unipd.it (L.F.); enrico.novelli@unipd.it (E.N.)
[3] Department of Agricultural, Food and Environmental Sciences, University of Perugia, 06126 Perugia, PG, Italy; sonia.esposto@unipg.it (S.E.); maurizio.servili@unipg.it (M.S.)
[4] Interdepartmental Centre for Industrial Agrofood Research, Alma Mater Studiorum—University of Bologna, 47521 Cesena, FC, Italy
* Correspondence: agnese.taticchi@unipg.it; Tel.: +39-075-585-7909

Academic Editor: Rubén Domínguez-Valencia

Received: 16 July 2025
Revised: 10 September 2025
Accepted: 12 September 2025
Published: 17 September 2025

Citation: Mercatante, D.; Balzan, S.; Esposto, S.; Barbieri, S.; Fontana, F.; Fasolato, L.; Rosa, V.D.; Servili, M.; Taticchi, A.; Novelli, E.; et al. Brine Enriched with Olive Wastewater Phenols: A Green Strategy to Reduce Nitrites in Cooked Ham. *Antioxidants* **2025**, *14*, x. https://doi.org/10.3390/xxxxx

Copyright: © 2025 by the authors. Submitted for possible open access publication under the terms and conditions of the Creative Commons Attribution (CC BY) license (https://creativecommons.org/licenses/by/4.0/).

Table S1. Cooking weight loss and technical yield.

	CP (%)	TY (%)
**C**	28.5	82.0
**S1**	28.1	79.2
**S2**	28.7	80.5
**S3**	29.2	79.2

CP: Cooking weight loss; TY: technical yield.
CP=[(weight after moulding – weight after cooking)/weight after moulding] x 100;
TY=(100-%injection) – [(100+%injection) x CP/(100)] [80].

Cooking loss and technical yield are important technological parameters. As expected, the absence of water-binding ingredients and other processing factors (such as tumbling techniques and cooking time) led to high water losses and reduced yields, which are not in line with commercial cooked ham values. Generally, a high-quality product has a yield of 98% and, in the case of products without polyphosphates, the cooking loss is 12-18%, up to 25% [97]. Pizza and Pedrielli [96] evaluated the effects of tumbling and cooking methods on cooked ham added with triphosphate, finding out product yields that varied between 87.7% and 102.7% and cooking loss between 11.8% and 19.2%.

Table S2. Microbial targets (CFU/g) of cooked ham samples after 0, 15 and 30 days of storage. C, Control (meat + maltodextrin + 150 mg of nitrites /kg of meat); S1, meat + 150 mg of nitrites /kg of meat + 200 mg phenols/kg of meat; S2, meat + 35 mg of nitrites /kg of meat + 200 mg phenols/kg of meat, S3, meat + 200 mg phenols/kg of meat.

		TVC	LAB	Yeast	Mould
T0	**C**	1.96±0.00	<LOD	<LOD	<LOD
	**S1**	1.48±2.09	<LOD	1.39±0.19	<LOD
	**S2**	2.41±0.64	2.94±0.29	1.40±0.18	<LOD
	**S3**	2.35±0.55	<LOD	<LOD	<LOD
T15	**C**	5.31±0.36	5.20±0.09	<LOD	<LOD
	**S1**	1.96±0.00	<LOD	<LOD	<LOD
	**S2**	2.81±0.03	2.84±0.45	<LOD	<LOD
	**S3**	2.01±0.44	<LOD	<LOD	<LOD
T30	**C**	<LOD	<LOD	3.04±0.18	<LOD
	**S1**	<LOD	1.48±2.09	<LOD	0.50±0.21
	**S2**	<LOD	7.03±0.03	<LOD	<LOD
	**S3**	<LOD	<LOD	<LOD	<LOD

Data are expressed as mean±standard deviation of three replicates; <LOD= lower than the limit of detection; TVC=Total Viable Count; LAB=Lactic Acid Bacteria.

Microbial analysis

As reported by Fasolato et al. [24], 10 g of sample were homogenized in a sterile stomacher bag with 90 mL of BPW (buffered peptone water) and then analyzed using appropriate decimal dilutions in Maximum Recovery Diluent (8 g of NaCl/L, 1 g of bacteriological peptone/L). Total viable count (TVC) was evaluated on Plate Count Agar (Biokar Diagnostics, ZAC de Ther, Allonne, Beauvais Cedex, France) incubated at 30 °C for 72 h. LAB (Lactic Acid Bacteria) was evaluated on MRSA agar (De Man, Rogosa and Sharpe agar, Biokar Diagnostics) incubated in anaerobic conditions at 30 °C for 48–72 h, while yeast and moulds were grown on Oxytetracycline Glucose Yeast Extract Agar (OGYE, Oxoid) at 25 °C for 3-5 days. The limit of detection considered in this study was <10 CFU/g. Qualitative analyses were also conducted to verify the presence of *Salmonella* spp. (ISO 6579-1) and *Listeria* spp. (ISO 11290-1).

The results were reported as log10 Colony Forming Units (CFU)/g of meat after the identification of the presumed colonies.

In general, the microbial contamination of ham showed a very low load of TVC (total viable count) throughout the entire storage period (Table S2). The critical level of spoilage microorganisms, such as lactic acid bacteria (LAB), was reached only at 30 days in S2 samples. Sensory decay of this product is typically observed when LAB levels exceed 7 Log CFU/g [98]. However, microbial contamination was sporadic and not consistently present in all the packaged products.

Table S3. Fatty acid profile of cooked ham samples after 0, 15 and 30 days of storage. C, Control (meat + maltodextrin + 150 mg of nitrites /kg of meat); S1, meat + 150 mg of nitrites /kg of meat + 200 mg phenols/kg of meat; S2, meat + 35 mg of nitrites /kg of meat + 200 mg phenols/kg of meat, S3, meat + 200 mg phenols/kg of meat.

Storage Time (days)	C		S1		S2		S3		SEM	*P* Form	St	Form*St
**C14:0**												
0	1.52	c,C	1.83	a,A	1.67	c,B	1.61	b,B	0.15	*NS*	***	*NS*
15	1.70	b,B	1.77	b,B	1.80	b,A	1.53	c,C				
30	1.85	a,B	1.68	c,C	2.03	a,A	2.01	a,A				
**C16:0**												
0	0.03	a,A	0.03	a,A	0.03	a,A	0.03	a,A	0.00	*NS*	***	*NS*
15	0.03	a,A	0.03	a,A	0.02	a,A	0.03	a,A				
30	0.02	a,A	0.02	a,A	0.02	a,A	0.03	a,A				
**C16:1**												
0	3.60	b,B	4.02	a,A	3.96	c,B	3.57	ab,B	0.38	***	*	***
15	3.50	b,AB	3.78	b,AB	4.73	b,A	3.05	b,B				
30	4.95	a,B	3.80	b,C	5.88	a,A	3.95	a,C				
**C17:0**												
0	0.25	a,A	0.19	b,B	0.28	a,A	0.17	a,B	0.00	*NS*	***	*NS*
15	0.27	a,A	0.25	a,A	0.20	b,B	0.19	a,B				
30	0.14	b,C	0.16	b,B	0.14	c,C	0.19	a,A				
**C18:0**												
0	13.03	ab,A	11.58	a,C	12.65	a,B	13.81	a,A	0.89	*NS*	***	*NS*
15	13.91	a,A	12.95	a,B	9.35	c,C	12.66	b,B				
30	12.22	b,A	11.63	b,B	10.38	b,C	11.54	c,B				
**C18:1 *n*-9**												
0	67.32	b,B	67.36	b,B	70.31	b,A	69.52	b,B	2.34	***	*	***
15	69.43	b,B	67.16	b,C	70.73	b,A	70.30	a,A				
30	75.58	a,A	70.33	a,C	72.42	a,B	70.48	a,C				
**C18:2**												
0	11.58	a,B	12.11	b,A	11.06	a,B	10.88	a,C	0.99	***	*NS*	**
15	11.32	a,A	11.38	c,A	10.74	b,B	10.14	a,B				
30	10.36	b,C	13.74	a,A	11.62	a,B	10.81	a,B				
**C18:3 *n*-3**												
0	0.87	b,B	0.98	a,A	0.90	a,AB	0.98	a,A	0.00	*	**	**

15	0.86	b,B	0.93	a,A	0.82	b,B	0.88	b,B				
30	0.92	a,A	0.90	a,AB	0.76	c,C	0.86	b,B				
C20:2												
	C		S1		S2		S3					
0	0.43	a,A	0.48	b,A	0.40	a,AB	0.37	b,B	0.00	***	*NS*	**
15	0.40	a,B	0.46	b,A	0.42	a,B	0.45	a,A				
30	0.48	a,B	0.59	a,A	0.45	a,B	0.44	a,B				
C20:3												
	C		S1		S2		S3					
0	1.38	a,B	1.42	a,A	1.47	a,A	1.06	a,C	0.01	*	**	**
15	0.59	b,D	1.29	b,A	1.19	c,B	0.76	b,C				
30	0.49	c,D	1.14	c,B	1.29	b,A	0.72	b,C				

Results as reported as means and standard error of the mean (SEM) of 3 independent replicates. a–c indicates significant differences (Tukey's test; $p \le 0.05$) within the same sample during the shelf-life. A-D indicate significant differences (Tukey's test; $p \le 0.05$) among treatments, *NS*, not significant; * $p<0.5$, ** $p<0.01$, *** $p<0.001$.

Table S4. Volatile organic compounds (expressed as µg/kg f.w. cooked ham of 4-methyl-2-pentanol equivalent) profile of cooked ham samples after 0, 15 and 30 days of storage. C, Control (meat + maltodextrin + 150 mg of nitrites/kg of meat); S1, meat + 150 mg of nitrites/kg of meat + 200 mg phenols/kg of meat; S2, meat + 35 mg of nitrites/kg of meat + 200 mg phenols/kg of meat, S3, meat + 200 mg phenols/kg of meat.

Storage Time (days)									SEM	*P*		
	Propanal									Form	St	Form*St
	C		S1		S2		S3		3.46	***	***	***
0	21.91	c,A	0.00	a,B	0.00	a,B	0.00	a,B				
15	30.32	b,A	0.00	a,B	0.00	a,B	0.00	a,B				
30	60.64	a,A	0.00	a,B	0.00	a,B	0.00	a,B				
	Pentanal											
	C		S1		S2		S3		10.28	***	***	***
0	177.60	c,A	20.81	c,C	8.42	c,D	34.26	c,B				
15	343.20	c,A	154.45	b,B	29.07	b,C	149.28	b,B				
30	540.94	a,A	145.46	a,C	81.17	a,D	227.72	a,B				
	Hexanal											
	C		S1		S2		S3		47.89	***	***	***
0	471.82	c,A	46.24	c,D	127.76	c,C	174.73	c,B				
15	1505.96	b,A	322.74	a,B	182.10	b,C	331.96	b,B				
30	2863.24	a,A	148.68	b,D	212.05	a,C	433.44	a,B				
	**Σ Aldehydes**											
	C		S1		S2		S3		134.98	***	***	***
0	649.42	c,A	67.05	c,D	136.18	c,C	208.99	c,B				
15	1879.48	b,A	477.19	a,B	211.17	b,C	481.24	b,B				
30	3464.82	a,A	294.14	b,C	293.22	a,C	661.16	a,B				
	Ethanol											
	C		S1		S2		S3		21.22	***	***	***
0	249.70	a,C	428.39	a,A	367.41	a,B	230.34	a,C				
15	95.09	c,B	60.67	c,C	39.38	c,D	212.88	b,A				
30	118.09	b,A	72.09	b,B	58.06	b,D	63.28	c,C				
	2-Methyl-2-buten-1-ol											
	C		S1		S2		S3		4.88	***	***	***
0	52.12	a,A	0.00	a,B	0.00	a,B	0.00	a,B				
15	22.18	c,A	0.00	a,B	0.00	a,B	0.00	a,B				
30	44.37	b,A	0.00	a,B	0.00	a,B	0.00	a,B				
	1-Pentanol											
	C		S1		S2		S3		5.34	***	***	***
0	151.35	a,A	0.00	b,C	0.00	a,C	47.63	a,B				
15	66.06	c,A	21.76	a,C	0.00	a,D	45.30	a,B				
30	132.12	b,A	0.00	b,C	0.00	a,C	43.52	a,B				
	Acetoin											
	C		S1		S2		S3		10.21	***	***	***

0	9.92	b,D	40.88	a,B	51.71	b,A	15.35	a,C				
15	24.02	a,C	33.85	b,B	49.91	c,A	14.36	a,D				
30	9.84	b,C	38.19	b,B	56.70	a,A	10.99	b,C				
1-Octen-3-ol												
	C		S1		S2		S3		17.34	***	***	***
0	111.72	b,A	0.00	b,B	0.00	a,B	0.00	b,B				
15	80.86	c,A	0.17	a,B	0.00	a,C	0.35	a,B				
30	161.72	a,A	0.00	b,C	0.00	a,C	0.35	a,B				
Terpinen-4-ol												
	C		S1		S2		S3		10.11	***	***	***
0	29.77	c,A	17.55	b,B	0.00	a,C	0.00	a,C				
15	32.56	b,A	0.00	c,B	0.00	a,B	0.00	a,B				
30	43.33	a,A	21.80	a,B	0.00	a,C	0.00	a,C				
**Σ Alcohols**												
	C		S1		S2		S3		78.90	***	***	***
0	604.59	a,A	469.27	a,B	486.82	a,B	293.32	a,C				
15	320.77	c,A	116.45	b,C	89.28	c,D	272.90	b,B				
30	509.47	b,A	132.08	b,B	114.76	b,C	118.14	c,C				
Methyl acetate												
	C		S1		S2		S3		3.99	***	***	***
0	88.81	a,B	98.17	a,A	86.39	a,B	57.19	a,C				
15	5.18	c,B	7.06	c,B	5.06	c,B	24.56	b,A				
30	6.80	b,B	3.55	b,C	3.37	b,C	10.75	c,A				
Ethyl acetate												
	C		S1		S2		S3		15.33	***	***	***
0	248.08	a,AB	293.88	a,A	211.34	a,B	228.22	a,B				
15	186.22	c,B	118.51	c,C	18.59	b,D	211.46	b,A				
30	206.63	b,A	165.81	b,B	20.85	b,C	216.17	b,A				
Isobutyl acetate												
	C		S1		S2		S3		45.09	***	***	***
0	554.24	a,B	795.23	a,A	491.98	a,C	0.00	a,D				
15	494.21	b,A	5.20	c,C	30.91	b,B	0.00	a,D				
30	360.21	c,B	628.21	b,A	10.41	c,C	0.00	a,D				
**Σ Esters**												
	C		S1		S2		S3		88.89	***	***	***
0	891.13	a,B	1187.29	a,A	789.72	a,C	285.41	a,D				
15	685.61	b,A	130.78	c,C	54.56	b,D	236.02	b,B				
30	573.64	c,B	797.58	b,A	34.63	c,D	226.93	c,C				
2-Butanone												
	C		S1		S2		S3		3.11	***	***	***
0	97.53	a,A	74.31	a,B	76.94	a,B	43.34	a,C				
15	19.95	ab,C	24.37	ab,B	5.39	c,D	34.29	b,A				

30	16.97	b,C	22.92	b,B	13.29	b,C	35.45	b,A				
4-Methyl-2-pentanone												
	C		S1		S2		S3		24.34	***	***	***
0	548.50	a,A	0.00	a,B	0.00	a,B	0.00	a,B				
15	179.90	c,A	0.00	a,B	0.00	a,B	0.00	a,B				
30	359.81	b,A	0.00	a,B	0.00	a,B	0.00	a,B				
**Σ Ketones**												
	C		S1		S2		S3		56.39	***	***	***
0	646.03	a,A	74.31	a,B	76.94	a,B	43.34	a,C				
15	199.85	c,A	24.37	b,C	5.39	c,D	34.29	b,B				
30	376.77	b,A	22.92	b,C	13.29	b,D	35.45	b,B				
Octane												
	C		S1		S2		S3		1.41	***	***	***
0	28.28	b,A	9.11	b,C	7.86	a,C	17.49	b,B				
15	25.33	c,A	19.76	a,B	5.02	b,D	8.71	c,C				
30	43.82	a,A	6.83	b,C	2.49	c,D	37.02	a,B				
Ethylbenzene												
	C		S1		S2		S3		75.23	***	***	***
0	413.11	a,B	285.91	b,D	368.94	a,C	829.64	a,A				
15	290.62	c,D	558.46	a,B	343.36	b,C	801.54	b,A				
30	353.94	b,B	227.31	c,C	331.02	c,B	785.91	c,A				
**Σ Hydrocarbons**												
	C		S1		S2		S3		156.87	***	***	***
0	441.39	a,B	295.02	ab,C	376.80	a,C	847.13	a,A				
15	315.95	c,C	578.22	a,B	348.38	ab,C	810.24	c,A				
30	397.76	ab,B	234.14	c,C	333.51	c,C	822.93	ab,A				
2-Ethylfuran												
	C		S1		S2		S3		1.34	***	***	***
0	12.10	c,A	0.00	a,B	0.00	a,B	0.00	a,B				
15	23.99	b,A	0.00	a,B	0.00	a,B	0.00	a,B				
30	47.97	a,A	0.00	a,B	0.00	a,B	0.00	a,B				
2-Penthylfuran												
	C		S1		S2		S3		12.65	***	***	***
0	63.95	a,A	55.01	a,B	0.00	a,C	0.00	a,C				
15	24.39	c,A	0.00	a,B	0.00	a,B	0.00	a,B				
30	48.79	b,A	0.00	a,B	0.00	a,B	0.00	a,B				
**Σ Furans**												
	C		S1		S2		S3		1.18	***	***	***
0	76.05	b,A	55.01	a,B	0.00	a,C	0.00	a,C				
15	48.38	c,A	0.00	b,B	0.00	a,B	0.00	a,B				
30	96.76	a,A	0.00	b,B	0.00	a,B	0.00	a,B				

Results as reported as means and standard error of the mean (SEM) of 2 independent replicates. a–c indicates significant differences (Tukey's test; $p \leq 0.05$) within the same sample during the shelf-life. A-D indicate significant differences (Tukey's test; $p \leq 0.05$) among treatments, *** $p < 0.001$.

References

24 Fasolato, L.; Carraro, L.; Facco, P.; Cardazzo, B.; Balzan, S.; Taticchi, A.; Andreani, N.A.; Montemurro, F.; Martino, M.E.; Di Lecce, G.; Toschi, T.G.; Novelli, E. Agricultural by-products with bioactive effects: A multivariate approach to evaluate microbial and physicochemical changes in a fresh pork sausage enriched with phenolic compounds from olive vegetation water. *Int. J. Food Microbiol.* **2016**, *228*, 34–43. https://doi.org/10.1016/j.ijfoodmicro.2016.04.003.

96 Pizza, A.; Pedrielli, R. Effetto delle Tecniche di Zangolatura e di Cottura sulla Resa e sull'Accettabilità del Prosciutto Cotto Ottenuto da Cosce di Diversa Qualità. *Ind Conserve* **2000**, *75*, 171–182.

97 Trevisani, M.; Balzan, S.; Cardazzo, B.; Fasolato, F.; Novelli, E. Prodotti di salumeria. In *Igiene e Tecnologie degli Alimenti*; Colavita, G. Ed.; Point Veterinaire Italie: Milano, Italy, 2023; pp. 329–374.

98 Kreyenschmidt, J.; Hübner, A.; Beierle, E.; Chonsch, L.; Scherer, A.; Petersen, B. Determination of the Shelf Life of Sliced Cooked Ham Based on the Growth of Lactic Acid Bacteria in Different Steps of the Chain. *J Appl Microbiol* **2010**, *108*, 510–520. https://doi.org/10.1111/j.1365-2672.2009.04451.x.

Disclaimer/Publisher's Note: The statements, opinions and data contained in all publications are solely those of the individual author(s) and contributor(s) and not of MDPI and/or the editor(s). MDPI and/or the editor(s) disclaim responsibility for any injury to people or property resulting from any ideas, methods, instructions or products referred to in the content.
